# The Cxcr2^+^ subset of the S100a8^+^ gastric granylocytic myeloid-derived suppressor cell population (G-MDSC) regulates gastric pathology

**DOI:** 10.3389/fimmu.2023.1147695

**Published:** 2023-09-08

**Authors:** Krystal D. Kao, Helmut Grasberger, Mohamad El-Zaatari

**Affiliations:** Division of Gastroenteorlogy, Department of Internal Medicine, University of Michigan Health System, Ann Arbor, MI, United States

**Keywords:** stomach, metaplasia, MDSC, gastritis, *Helicobacter pylori*, *Helicobacter felis*

## Abstract

**Introduction:**

Gastric myeloid-derived suppressor cells (MDSCs) are a prominent population that expands during gastric pre-neoplastic and neoplastic development in humans and mice. However, the heterogeneity of this population has circumvented the ability to study these cells or understand their functions. Aside from Schlafen-4^+^ (Slfn-4^+^) MDSCs in mouse studies, which constitute a subset of this population, limitations exist in characterizing the heterogeneity of the gastric CD11b^+^Ly6G^+^ population and targeting its different subsets. Here we identify S100a8 as a pan-specific marker for this population and utilize it to study the role of the S100a8^+^Cxcr2^+^ subset.

**Methods:**

We profiled gastric CD11b^+^Ly6G^+^ versus CD11b^+^Ly6G^-^ myeloid cells by transcriptomic and single-cell RNA sequencing. We identified S100a8 as a pan-specific marker of the gastric granulocytic MDSC (G-MDSC) population, and generated S100a8^Cre^Cxcr2^flox/flox^ to study the effects of Cxcr2 knockdown.

**Results:**

Following 6-months of *Helicobacter felis* infection, gastric CD11b^+^Ly6G^+^ G-MDSCs were highly enriched for the expression of S100a8, S100a9, Slfn4, Cxcr2, Irg1, Il1f9, Hcar2, Retnlg, Wfdc21, Trem1, Csf3R, Nlrp3, and Il1b. The expression of these distinct genes following 6mo *H. felis* infection marked heterogeneous subpopulations, but they all represented a subset of S100a8^+^ cells. S100a8 was identified as a pan-marker for CD11b^+^Ly6G^+^ cells arising in chronic inflammation, but not neutrophils recruited during acute gut infection. 6mo *Helicobacter felis*-infected S100a8^Cre^Cxcr2^flox/flox^ mice exhibited worsened gastric metaplastic pathology than Cxcr2^flox/flox^ mice, which was associated with dysregulated lipid metabolism and peroxidation.

**Conclusion:**

S100a8 is a pan-specific marker that can be used to target gastric G-MDSC subpopulations, of which the Cxcr2^+^ subset regulates gastric immunopathology and associates with the regulation of lipid peroxidation.

## Introduction

Gastric MDSCs were originally described to comprise a heterogeneous and immature myeloid cell population, which becomes expanded in the metaplastic and neoplastic gastric microenvironment of human patients and mouse models (1–4). However, the heterogeneity of these cells, in humans and mice, has presented an obstacle to our understanding of their functions. Gastric MDSC expansion was first modeled in mice by overexpressing IL-1β specifically in their stomach, which was associated with the development of dysplastic and neoplastic lesions (1). We later uncovered a population of gastric myeloid cells that specifically expressed Schlafen-4 (Slfn4), representing a subset of gastric CD11b^+^Gr1^+^ cells, which was expanded in the inflammatory milieu of metaplastic gastric lesions following 6-month *Helicobacter felis* (*H. felis*) infection (5). This population was later identified to represent gastric granulocytic MDSCs (G-MDSCs) (2). Lin et al. showed that these Slfn4^+^ myeloid cells elicit T cell inhibitory activities, therefore corroborating the myeloid-derived suppressor function of gastric CD11b^+^Gr1^+^ cells towards T cells (2). In human pre-neoplastic and carcinogenic lesions, an expansion of G-MDSCs expressing the Slfn4 homolog, Slfn12l, occurs (6). However, the heterogeneity of the human gastric G-MDSC population, marked in humans by CD11b, CD15, and CD33 (2, 7), which elicits myeloid-derived inhibitory effects on T cells, presents an obstacle to dissecting the variety of functions of these cells.

While the T cell-inhibitory activity of MDSCs on T cells has been established, their functional diversity due to their heterogeneity remains unclear, and a mechanism to study these heterogeneous sub-populations remains elusive. Learning about their functional diversity in mouse models is necessary, as it can provide insight into the diverse roles of the heterogenous G-MDSC sub-populations in human disease. Nevertheless, the characterization of the Slfn4^+^ populations in mice and Slfnl2l^+^ populations in humans provided one successful approach to study a specific subset of the heterogenous granulocytic MDSC populations. Here, we address the unmet need of characterizing the heterogeneity of the murine gastric CD11b^+^Ly6G^+^ granulocytic MDSC population in mice, characterizing specific markers for its sub-populations, and identifying a pan-specific marker to target these sub-populations. The study in mice provides an avenue to model the functions in which these heterogeneous populations operate and can elicit variable concomitant effects on gastric pathology.

## Materials and methods

### Mice

All animals were housed in the animal maintenance facility at the University of Michigan Health System. This research was undertaken with the approval of the Committee on Use and Care of Animals at the University of Michigan under protocol #PRO00009914. Mouse genotypes were confirmed by PCR using mouse tails. Mouse strains (1) S100a8^Cre^ #021614 (2), Cxcr2^flox/flox^ #24638, and (3) C57BL/6J #000664 were all obtained from Jackson Labs (Bar Harbor, ME). S100a8^Cre^Cxcr2^flox/flox^ were generated by crossing S100a8^Cre^ mice to Cxcr2^flox/flox^ mice. Mice were housed under specific pathogen-free conditions and fasted overnight before use, with free access to water.

### *Helicobacter felis* infection

*H. felis* (CS1 strain) stocks were stored in 50% glycerol solution at −80°C. Bacteria were cultured in sterile-filtered Brucella broth (BD, Franklin Lakes, NJ) plus 10% FBS (Atlanta Biologicals, Lawrenceville, GA) using the GasPak™ EZ Campy Container System (BD) at 37°C with 150 rpm shaking. The cultures were spun down at 2700 rpm at room temperature, and the pellets resuspended in Brucella broth plus 10% FBS (Thermo Fisher Scientific, Houston, TX). Cells were counted using a hemocytometer by diluting the cells 1:100 in 9:1 HBSS/Formalin solution. Mice were gavaged 3 times over 3 days with 10^8^ *H. felis* cells in 100 μl of Brucella broth. Mice were infected for 6 months prior to euthanasia.

### Antibiotic administration and infection with *C. difficile*

Antibiotic administration and infection with *C. difficile* was performed as described previously (8). *C. difficile* strain VPI 10463 (ATCC 43255) was kindly provided by Garry B. Huffnagle. Spores were prepared as described previously (9). Cefoperazone (0.5 mg/ml; MP Bioworks; cat. #199695) was given to mice in sterile drinking water for 5 days. Mice were then switched to regular water for 2 days before being orally gavaged with 6 × 10^6^ C*. difficile* spores. Mice were then euthanized after 2 d of infection.

### Tissue collection, processing and immunohistochemistry

Stomach tissue collection was performed as described previously (10). Briefly, the stomachs were opened along the greater curvature. For histology, gastric strips from both the lesser and greater curvatures were fixed in formalin for paraffin sections. For DNA, gastric tissue was snap frozen in liquid nitrogen and extracted using the DNEasy Blood and Tissue Kit (Qiagen, Valencia, CA). For flow cytometry, gastric cells were digested using a modified version of the protocol described by Geem et al. (11), which utilizes 17.9 μg/ml Liberase TM (Cat #05401119001, Roche Diagnostics Corporation, Indianapolis, IN) instead of Type VIII collagenase. Immunohistochemistry and immunofluorescence were performed as described previously (10), using antibodies against the following targets: calprotectin (#ab22506, Abcam); CD11b-AF594 (clone M1/70, #101254, BioLegend); Cxcr2-AF488 (#FAB2164G, R&D Systems); GSII-FITC lectin (#FL-1211, Vector Labs, Burlingame); H^+^/K^+^-ATPase-β (#D032-3, Medical and Biological Laboratories, Woburn, MA); intrinsic factor (gift from David Alpers, Washington University, St. Louis, MO); E-cadherin-FITC (#612130, BD Biosciences, San Jose, CA); E-cadherin-AF647 (#560062, BD Biosciences); and 4 Hydroxynonenal antibody (#ab46545, Abcam).

### Fluorescence-activated cell sorting

FACS was performed as described previously (10), using FACSAria III (BD, Franklin Lakes, NJ). Live cells were gated using LIVE/DEAD Aqua Stain (cat #L34957; Life Technologies, Grand Island, NY). For the quantification of immune cell frequencies within the gastric mucosae, the cells were stained with the following antibodies: (i) for myeloid cells: CD11b-eFluor 450 (clone M1/70, cat #48-0112-82; eBioscience, San Diego, CA) and Ly6G-PE (clone 1A8, cat. #127607; BioLegend, San Diego, CA); (ii) for T cells: CD4-FITC (clone GK1.5, cat. #11-0041-85, eBioscience) and CD8-PerCpCy5.5 (clone 53-6.7, cat. #45-0081, eBioscience); (iii) for Natural Killer cells: NK1.1-PECy5 (clone PK136, BioLegend); and (iv) for B cells: B220-PE-Cy7 (clone RA3-6B2, cat. #103221, BioLegend) and IgM-PE (clone eB121-15F9, cat. #12-5890, eBioscience).

For the characterization of gastric myeloid immune cell overlap using 7-color FACS, dissociated gastric cells were stained with: (i) LIVE/DEAD Aqua (cat #L34957; Life Technologies), (ii) CD11b-eFluor 450 (clone M1/70, cat #48-0112-82; eBioscience), (iii) Ly6G-PE (clone 1A8, cat. #127607; BioLegend), (iv) Ly6C-APC-Cy7 (clone HK1.4, cat #128025, BioLegend), (v) F4/80-PerCpCy5.5 (clone BM8, cat #45-4801-80, eBioscience), (vi) CD103-APC (clone 2E7, cat #17-1031-82, eBioscience), and (vii) CD11c-PeCy5 (clone N418, cat #15-0114-81, eBioscience).

### Microarray analyses of FACS-sorted cells

FACS-sorted cells from the stomach were collected in PBS + 2% FBS, centrifuged at 300 x g, and the pellet suspended in buffer RLT plus β-ME (cat. #74004, RNEasy Microkit, Qiagen). The mixture was dissociated using QIAshredder columns (cat. #79654, Qiagen), and RNA extracted using the RNEasy Microkit (cat. #74004, Qiagen). Microarray was performed by the University of Michigan DNA Advanced Genomics Core using the NuGEN Technology (NuGEN Technologies, Inc., San Carlos, CA). Expression values were calculated for each gene using a robust multi-array average (RMA) (12), which is a modeling strategy that converts the PM probe values into an expression value for each gene. As a quality control step, a principal components analysis (PCA) was fit on the expression values, and the first two principal components were plotted. Samples with similar expression profiles were validated to group near each other. For analyses, the list of probesets were limited to those annotated as ‘main’ by Affymetrix. Other probesets included internal controls and were mostly not annotated. Probesets that had a 2-fold or greater change were selected, with the added constraint that the average expression value of one of the two groups was 2^3^ or greater. This prevented the selection of large fold changes based on two small numbers. Selected genes were output in HTML tables which contained both probeset-specific information (probeset ID, statistics, expression values), and gene-specific information (gene name, symbol, links to online databases). The entire expression values were also output in a text file that could be opened in excel for further analyses.

### Single cell RNA sequencing

Gastric fundus/corpus tissue was first digested with EDTA, and then further digested using a modified version of the protocol described by Geem et al. (11), which utilizes 17.9 μg/ml Liberase TM (Cat #05401119001, Roche Diagnostics Corporation) instead of Type VIII collagenase that was described in their initial protocol. The digested cells from the EDTA and liberase digestions were pooled together. Live cells were enriched using EasySep Dead Cell Removal (Annexin V) Kit (STEMCELL Technologies Inc., Cambridge, MA). Sc-RNASeq was performed by the University of Michigan DNA Advanced Genomics Core, with libraries constructed and subsequently subjected to 151 paired end cycles on the NovaSeq-6000 platform (Illumina). Bcl2fastq2 Conversion Software (Illumina) was used to generate de-multiplexed Fastq files. Mapping and quantitation were also done by the AGC, using the mm10-2020-A mouse reference. Cell Ranger was used to generate sequence alignment (bam) files, and feature-barcode matrices for individual samples, to aggregate the samples and generage aggregated feature-barcode matrices, and to cluster the cells and generate “cloupe” files, for import into the Loupe Browser. The data was analyzed using cloupe browser by 10x Genomics (Pleasanton, CA).

### scRNA-seq velocity plot and partition-based graph abstraction plot generation

The default UMAP of aggregated data gave 36 clusters, including a visually distinct set of two overlapping clusters that were determined to be composed of myeloid cells. This Myeloid set was extracted as a single cluster for subclustering and trajectory analysis. Velocity (13) was calculated and plotted on the UMAP projection of the Myeloid Cluster in each of the four individual samples. To produce the velocity plots, velocyto (13) (v. 0.17.17) and scVelo (14) (v. 0.0.4, with Python 3.7.12) were used as follows: The UMAP coordinates and barcodes were exported from the Loupe Browser for the aggregated myeloid cluster. Then, for each sample, velocyto was run from the command line with the sample UMAP coordinates, a gtf file containing positions of repetitive elements to mask, the position-sorted bam file of filtered raw reads, and the mouse gtf file “Mus musculus.GRCm38.98.gtf”. The output is a file in “loom” format, designed to efficiently store single-cell datasets and metadata.

The loom files were input into an R (v. 4.1.3) with the package “reticulate” (v. 1.25) to run Python in R. The Python package “scVelo” was imported to calculate the cellular dynamics. For each sample, using the UMAP projection coordinates and the calculated velocity, with ggplot2 (v 2.3.4) library loaded, a UMAP plot could be produced with the rate and direction streams. The Myeloid Cluster was then reclustered using Seurat (v. 4.1.0), starting from the aggregated data with default parameters, but specifying 10 principal components and a resolution of 0.1, giving four sub-clusters. Significant positive marker genes for each sub-cluster were identified with Seurat’s “FindAllMarkers”. To prepare for computing the velocity for the aggregated data, the loom files from each of the individual samples were subset to include only cells from the Myeloid Cluster, then converted to a Annotated Data Frames including cell ID’s annotated with sub-cluster membership (since bam files are not available for the aggregated Cell Ranger output). These were concatenated into a single Annotated Data Frame for input into scVelo, resulting in velocity calculations for the aggregated sea, calculated as described above and displayed using the original UMAP projections. The same sub-clustering and velocity calculation was repeated using a resolution of 0.2 in the Seurat clustering step, giving 7 clusters. For both the sub-clustering plus velocity sets of results, PAGA (“Partition-based graph abstraction”) (15) was performed to infer trajectory inferrence and lineage relationships. The sub-clusters and trajectories are shown projected onto the original UMAP.

### Pathological scoring of metaplasia

The criteria for detecting SPEM were described previously (16, 17). SPEM was quantified by immunofluorescent co-localization of GSII and intrinsic factor (IF). Four strips of gastric mucosa for each mouse (2 from lesser and 2 from greater curvatures) were sectioned in paraffin blocks and stained with GSII and IF. 10x images were captured spanning the entire sections by confocal microscopy. The percentage area of metaplasia was calculated by dividing the number of 10x focal planes containing metaplasia over the total number of captured 10x focal planes spanning the entire fundic/corpus section for each mouse. The analysis was performed over the entire sections spanning 4 histological strips (2 from lesser and 2 from greater curvatures) for each mouse.

### *H. felis* DNA quantification

Gastric tissue from the corpus and fundus was snap frozen and stored at −80°C. Total DNA was extracted using the DNEasy Blood and Tissue Kit (Qiagen). Quantitative PCR was performed using the *H. felis* Fla-B primers, Forward: 5′ TTCGATTGGTCCTACAGGCTCAGA 3’, Reverse: 5′TTCTTGTTGATGACATTGACCAACGCA 3′ on a CFX96 real-time PCR detection system (Bio-RAD).

### Statistical analysis

Data were tested for normality using the Shapiro-Wilk W test (Prism, GraphPad Software, La Jolla, CA). Data were compared using one-way analysis of variance (ANOVA) with Dunnet’s (parametric) or Dunn’s (non-parametric) multiple comparison tests (Prism). P values less than 0.05 were considered significant.

## Results

### Gastric MDSCs display a distinctive transcriptional profile relative to gastric non-MDSC myeloid cells

**Figure 1:**
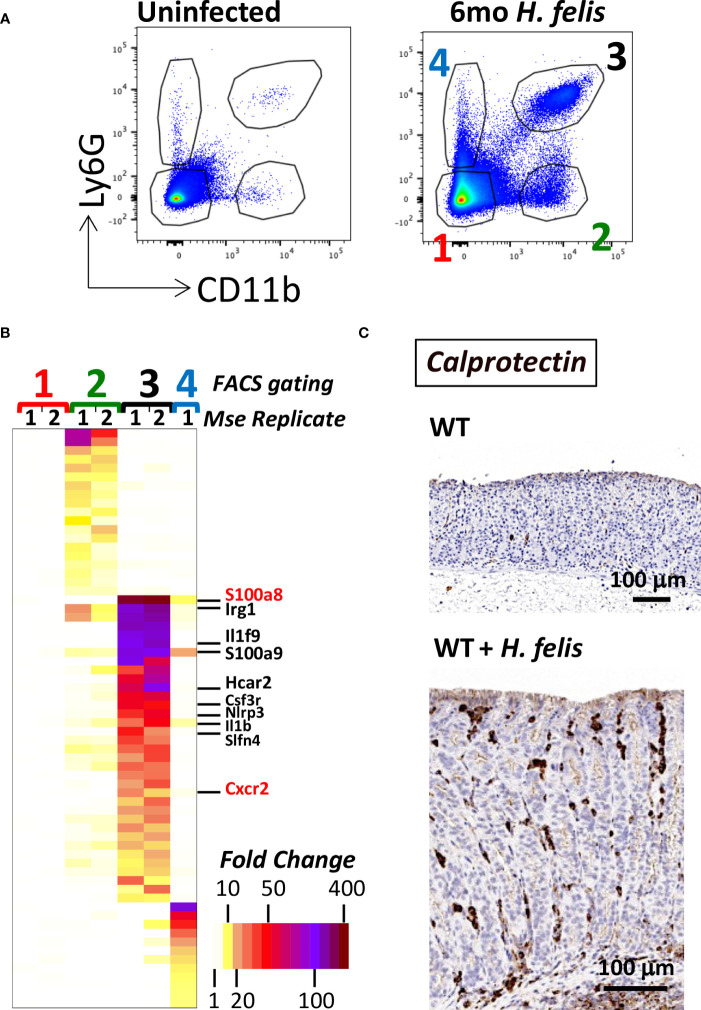
Gastric G-MDSCs are characterized by a specific transcriptional signature and can be identified by calprotectin (S100a8 and/or S100a9) expression. **(A)** Representative FACS plots of gastric MDSC versus non-MDSC myeloid cell populations using CD11b and Ly6G cell-surface protein markers, of dissociated gastric cells from uninfected versus 6mo *H felis*-infected WT mouse stomachs. Red font “1” annotates gastric non-myeloid and Ly6G-negative cells. Green font “2” annotates CD11b^+^Ly6G^-^ non-G-MDSC myeloid cells. Black font “3” annotates gastric CD11b^+^Ly6G^+^ G-MDSC myeloid cells. Blue font “4” annotates gastric CD11b^-^Ly6G^+^ non-myeloid cells. **(B)** Microarray heatmap showing the highest expressed genes that are specifically enriched in gastric CD11b^+^Ly6G^-^ non-G-MDSC myeloid cells (annotated as green font “2”), versus those specifically enriched in gastric CD11b^+^Ly6G^+^ G-MDSC myeloid cells (annotated as black font “3”), and those specifically enriched in non-myeloid gastric CD11b^-^Ly6G^+^ control cells (annotated as blue font “4”). The gene names for the calprotectin subunits S100a8 and S100a9, Il1b, Slfn4, and Cxcr2 are listed (S100a8 and Cxcr2 are annotated in red font because they will later be used in the study for Cre/Flox generation). **(C)** Representative image of the immunohistochemical staining of calprotectin (brown) in 6mo *H felis*-infected mouse gastric mucosa relative to uninfected.

To gain insight into the differences between gastric G-MDSCs versus other non-G-MDSC myeloid populations, we FACS sorted gastric myeloid cells, from 6mo *H. felis*-infected stomachs, using the previously characterized mouse markers CD11b^+^Ly6G^+^ (for G-MDSCs) and CD11b^+^Ly6G^-^ (for non-G-MDSC myeloid cells) (1) (**Figure 1A**). Microarray analyses showed distinctive transcriptional profiles for G-MDSCs versus non-G-MDSCs relative to other non-myeloid gastric cells (**Figure 1B**; **Supplementary Figures 1, 2**). In particular, gastric G-MDSCs expressed high levels of the calprotectin subunits S100a8 and S100a9, Il1b, Cxcr2, and our previously identified MDSC marker, Schlafen-4 (Slfn4) (5) (**Figure 1B**). Additional highly expressed genes are listed in **Figure 1B** and **Supplementary Figure 2**. In contrast, non-G-MDSC gastric myeloid cells highly expressed synaptic nuclear envelope protein 1 (Syne1) and serpin peptidase inhibitor B2 (Serpinb2) (**Supplementary Figure 2**). Non-myeloid CD11b^-^Ly6G^-^ cells did not express any of the aforementioned markers, while CD11b^-^Ly6G^+^ cells comprised a mixture of B and T cells based on their expression markers (**Supplementary Figure 2**). As we observed highly specific expression of the calprotectin subunits, S100a8 and S100a9 in CD11b^+^Ly6G^+^ G-MDSCs (**Figure 1B**), we utilized these markers to spatially visualize gastric G-MDSCs. We stained paraffin sections from the stomachs of 6mo *H. felis*-infected versus uninfected mice with calprotectin (S100a8/S100a9) antibodies (**Figure 1C**). Calprotectin-expressing cells constituted a subset of gastric CD11b^+^ myeloid cells in the chronically inflamed gastric mucosa as shown by immunofluorescent staining (**Supplementary Figure 3**). Hence, we showed that gastric G-MDSCs are distinct from non-G-MDSC myeloid cells and can be readily identified by calprotectin (*i.e.*, S100a8 and/or S100a9) expression.

### Specificity of S100a8 and S100a9 expression to gastric G-MDSCs relative to gut neutrophils in acute infection

**Figure 2:**
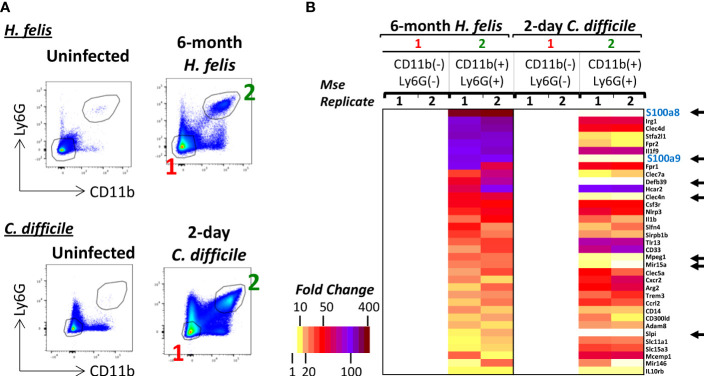
S100a8 and S100a9 are enriched in gastric G-MDSCs but not in acutely recruited gut neutrophils. **(A)** Representative FACS plots of gastric G-MDSCs versus cecal neutrophils in 6-month *H felis*-infected stomachs versus 2-day *C difficile* infected ceca respectively. **(B)** Microarray heatmap showing the highest expressed genes that are specifically enriched in gastric CD11b^+^Ly6G^+^ G-MDSCs relative to CD11b^+^Ly6G^+^ cecal neutrophils in 6-month *H felis*-infected stomachs versus 2-day *C difficile* infected ceca respectively. The black arrows annotate the genes that are highly expressed in G-MDSCs of 6 month *H felis*-infected stomachs but not in neutrophils of 2-day *C difficile* infected ceca.

We sought to evaluate the specificity of S100a8 and S100a9 expression in gut CD11b^+^Ly6G^+^ G-MDSCs, which arise during long-term chronic infection, versus gut CD11b^+^Ly6G^+^ neutrophils, which are rapidly recruited during acute infection. Due to the lack of existence of acute infection models for the murine stomach that lead to rapid neutrophilic infiltration, which would be sufficient for FACS isolation, we compared the transcriptional profiles of CD11b^+^Ly6G^+^ MDSCs from chronic 6mo *H. felis* infected stomachs versus CD11b^+^Ly6G^+^ neutrophils from acute 2-day *C. difficile*-infected ceca (**Figure 2**). Transcriptomic analyses showed that high S100a8 and S100a9 expression was specific to gastric G-MDSCs from chronic infection, but not acutely recruited gut neutrophils (**Figure 2**). Other genes that were specific to G-MDSCs, but were absent in acutely recruited neutrophils, are also annotated in **Figure 2**.

### S100a8 is a pan marker of gastric G-MDSCs that comprises heterogeneous subsets of gastric G-MDSC subpopulations expressing different markers

**Figure 3:**
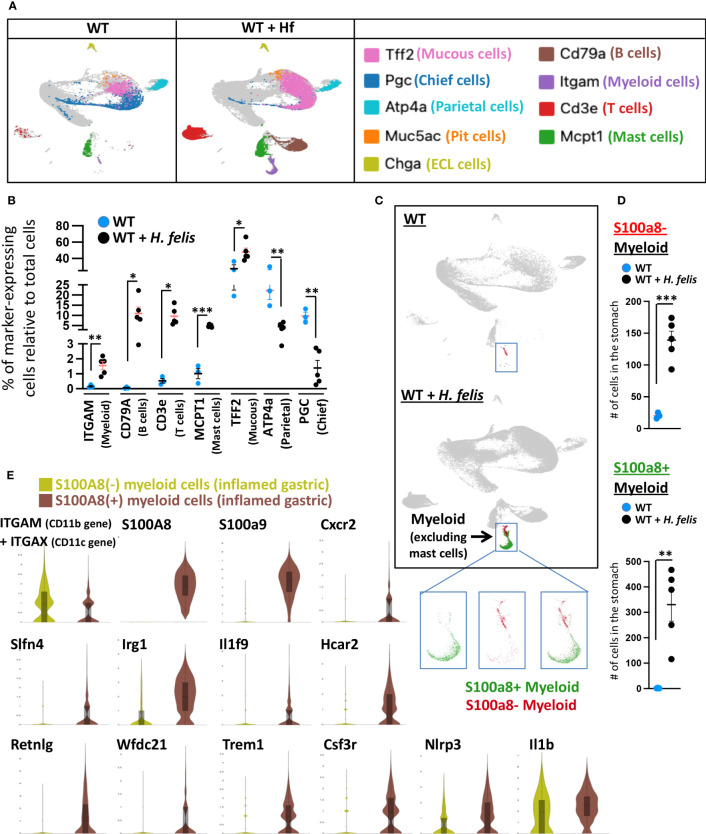
S100a8-expressing cells recapitulate the G-MDSC microarray transcriptional signatures on a single cellular level. **(A)** Representative UMAP plots of gastric dissociated cells from 6mo *H felis*-infected gastric mucosa relative to uninfected stomach (individual mouse scRNA-Seq plots are shown in **Supplementary Figure 4**). Markers of different individual populations are color-coded and annotated on the right. **(B)** Quantification by scRNA-Seq of the percentage of total gastric cells expressing specific markers of different immune or epithelial cells, in five 6mo *H felis*-infected mouse stomachs relative to three uninfected mouse stomach controls. Each data point represents one mouse. **(C)** Representative UMAP plots showing the expansion of gastric S100a8^+^ versus S100a8^-^ myeloid cells following 6-month *H felis* infection. The blue inset marks gastric myeloid cells excluding mast cells (based on the characterization in **(A)**). Three inset panels are shown, with the left panel displaying the localization within the cluster of S100a8^+^ cells on their own (green), the middle panel displaying the S100a8- cells on their own (red), and the right panel displaying the localization within the cluster of both S100a8^+^ (red) and S100a8^-^ (green) cells. **(D)** Quantification by scRNA-Seq of the number of gastric S100a8^+^ (*upper panel*) and S100a8^-^ myeloid cells (*lower panel*) following 6-month *H felis* infection. **(E)** Violin plot comparisons, from pooled 4 mice per group, of the genes enriched in gastric S100a8^+^ myeloid cells relative to S100a8^-^ myeloid cells in the 6-month *H felis*-infected gastric mucosa, which coincides with the list of genes identified by the microarray in **Figure 1B**, but additionally detects Retnlg, Wfdc21, and Trem1. Each data point represents one mouse. Error bars = means +/- SEM. ***P < 0.001; **P < 0.01; *P < 0.05.

To confirm the validity of calprotectin as a gastric G-MDSC marker, we compared the expression profiles of S100a8^+^ versus S100a8^-^ myeloid cells obtained from single-cell RNASeq of the 6mo *H. felis*-infected mouse stomach. Total gastric cells from uninfected and *H. felis*-infected mice were clustered in an unsupervised manner, as shown in **Supplementary Figure 4** and **Supplementary Table 1**, based on which the total gastric myeloid cell population (non-mast cell) was identified. This cluster was validated by positive Itgam (CD11b gene) expression (**Figure 3A**; **Supplementary Figure 5**; **Supplementary Table 2**), and was accordingly marked by the boxed blue inset in **Figure 3C**. The frequency of these Itgam-expressing myeloid cells was increased upon *H. felis* infection as determined by scRNA-Seq of the 5 stomachs of H*. felis*-infected mice relative to 3 uninfected controls (**Figures 3A, B**; **Supplementary Figure 5**; **Supplementary Table 2**). Changes in other immune (B cell, T cell, and mast cell) and epithelial (mucous and parietal) cell markers upon *H. felis* infection were also validated by scRNA-Seq of the stomachs of these mice, which also corroborated mucous cell expansion and parietal cell loss (**Figures 3A, B**; **Supplementary Figure 5**; **Supplementary Table 2**). We observed, by scRNA-Seq clustering, 2 distinct populations of S100a8^+^ versus S100a8^-^ myeloid cells (**Figure 3C**, *inset*, and **Figure 3D**), which were both increased after 6mo *H. felis* infection (**Figure 3D**; **Supplementary Figure 6**). Consistent with the microarray results, and despite the low level of detectability of Ly6G gene expression by scRNA-Seq at the sequencing depth we performed, Ly6G gene expression was only detected in CD11b^+^S100a8^+^ but not in CD11b^+^S100a8^-^ myeloid cells (**Supplementary Figure 7**), therefore providing evidence for the specificity of Ly6G expression within S100a8^+^ myeloid cells. Comparison of the gene signatures of S100a8^+^ versus S100a8^-^ cells by single cell RNASeq (**Figure 3E**; **Supplementary Figure 8**) corroborated the transcriptional enrichment of identical markers to those specifically identified in gastric G-MDSCs by microarray (*please compare these common markers in* **Figure 3E** *and* **Supplementary Figure 8** *to those in* **Figure 1B**). The myeloid sub-populations expressing the markers we identified in **Figure 1B** (Cxcr2, Slfn4, Irg1, Il1f9, Hcar2, Csf3R, and to a lesser extent Nlrp3 and/or Il1b) represented subsets of the S100a8^+^ population (**Figure 3E**; **Supplementary Figure 8B**), demonstrating that the S100a8 marker represented a pan-specific marker for gastric G-MDSCs encompassing all the other markers. Single-cell RNA-Seq further identified 3 additional markers, which had not been detected by microarray, and which were highly expressed by gastric S100a8^+^ cells including Resistin-like gamma (Retnlg), WAP four-disulfide core domain 21 (Wfdc21), and Triggering receptor expressed on myeloid cells 1 (Trem1) (**Figure 3E**; **Supplementary Figure 8**). However, the expression of the markers we identified in our myeloid subpopulations did not represent distinct myeloid cell populations, as the cellular populations expressing these markers overlapped (**Supplementary Figure 9**). This indicated that while S100a8^+^ myeloid cells encompassed the majority of the gastric G-MDSC population, the heterogeneous subsets of this population, which expressed variable markers, were overlapping. However, we identified one gastric myeloid cell sub-population expressing S100a8^+^Irg1^+^Slfn4^+^Trem1^+^Csf3r^+^Nlrp3^+^Il1b^+^, which expressed all our identified gastric G-MDSC markers at higher levels than the S100a8^+^ subpopulation that did not express all of these markers at the same time (**Supplementary Figure 10**). We conclude that S100a8 is a pan marker of G-MDSCs, with identical enriched markers by microarray and scRNA-Seq, although these enriched markers do not constitute distinct, but rather overlapping, populations.

### Differentiation trajectories of gastric S100a8^+^ versus S100a8^-^ myeloid cell populations

Velocity and PAGA plots were generated to determine the differentiation trajectories of gastric myeloid S100a8^+^ versus S100a8^-^ cell populations. This data identified distinct progenitors for gastric myeloid S100a8^+^ versus S100a8^-^ cells (**Supplementary Figures 11A, B**; **Supplementary Table 3**). The myeloid S100a8^+^ progenitors were enriched in S100a8, B cell markers CD79a and CD79B, and the T cell marker CD3G expression (**Supplementary Figure 11**; **Supplementary Table 3**), whereas the myeloid S100a8^-^ progenitor cells were enriched with the expression of F13A1, FN1, and S100A4 (**Supplementary Figure 11**; **Supplementary Table 3**). This indicated that the G-MDSC and non-G-MDSC gastric populations might arise from distinct projenitoris, and may not directly give rise to each other. It remains unclear why the S100a8^+^ myeloid progenitor cells expressed lymphoid markers, which requires future analyses. It is important to note that B cell marker CD79A expression had been previously reported on immature MDSCs (18), and CD3 had been described to be expressed by myeloid cells (19). These newly identified progenitor cells will be subject of future study.

### Cxcr2-expressing gastric myeloid cells constitute a subset of S100a8^+^ gastric G-MDSCs

**Figure 4:**
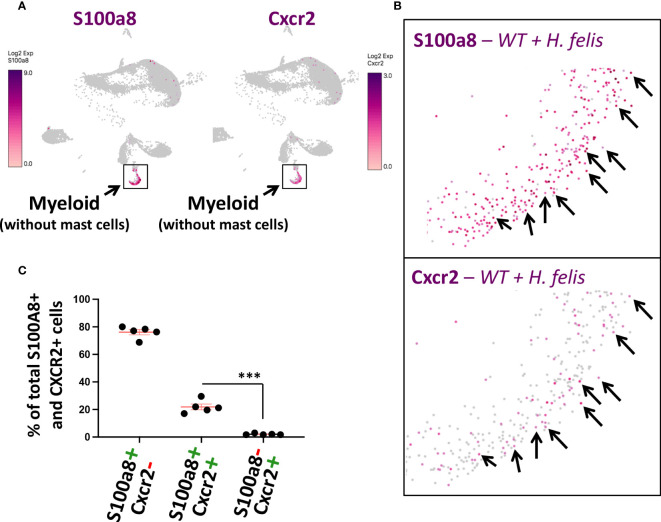
Gastric Cxcr2^+^ cells represent a subset of S100a8^+^ gastric G-MDSCs. **(A, B)** Comparison of S100a8 and Cxcr2 single cell expression patterns using UMAP plots of gastric scRNA-Seq data. Black arrows annotate single cells in which both S100a8 and Cxcr2 are co-expressed. **(C)** Percentages of gastric S100A^+^Cxcr2^+^ cells in comparison to S100A^+^Cxcr2^-^ cells and S100A^-^Cxcr2^-^ cells in 6mo *H felis*-infected mice, as determined by scRNA-Seq. Each data point represents one mouse. Error bars = means +/- SEM. ***P < 0.001.

Since Cxcr2 had previously been shown to play a necessary role in MDSC differentiation (20) and/or recruitment (21), thereby representing a functionally important molecule that can regulate MDSC function, and since we have detected Cxcr2 to be highly expressed in gastric G-MDSCs by microarray and by single-cell RNASeq (**Figures 1B**, **3E**; **Supplementary Figures 2, 8**), we sought to employ S100a8^Cre^, which encompasses the gastric G-MDSC population, as a driver to knock down Cxcr2 within the S100a8^+^Cxcr2^+^ subpopulation of gastric G-MDSCs. This was to be utilized as a proof-of-concept to validate the adequacy of the S100a8 promoter in targeting one of the G-MDSC subpopulations we identified, and also as a means to gain insight into one of the heterogenous aspects of the functions of G-MDSCs in gastric pathology. For this purpose, and to determine whether a Cre/Flox strategy would be appropriate for depleting Cxcr2 from S100a8^+^ G-MDSCs, we first determined the proportion of gastric S100a8^+^ G-MDSCs that expressed Cxcr2, and whether Cxcr2 was specific to gastric G-MDSCs as the microarray scRNA-Seq experiments indicated. ScRNASeq showed that Cxcr2^+^ myeloid cells represented a subset of S100a8^+^ G-MDSCs during chronic *H. felis* infection (**Figures 4A, B**, *black arrows*; and **Figure 4C**). Therefore, we sought to utilize the S100a8 promoter to cell-specifically ablate Cxcr2 from MDSCs, in attempt to determine the effect of MDSC-specific Cxcr2 ablation on chronic *H. felis*-induced gastric pathology.

Before generating the S100a8^Cre^Cxcr2^f/f^ mice, we also investigated the cell types which produced the Cxcr2 ligands, which comprised Cxcl1, Cxcl2, Cxcl3, and Cxcl5 (**Supplementary Figure 12**). Cxcl1 and Cxcl5 production were induced in epithelial cells following chronic *H. felis* infection, while Cxcl2 and Cxcl3 production was induced in epithelial, myeloid, and T cell subsets following infection (**Supplementary Figure 12**). Therefore, while Cxcr2 was specific to a subset of G-MDSCs within the inflamed gastric mucosa, the ligands for the Cxcr2 receptor were produced by multiple sources including epithelial, myeloid and T cells.

### S100a8^+^ cell-specific Cxcr2 ablation exacerbates chronic *H. felis* gastric immunopathology

**Figure 5:**
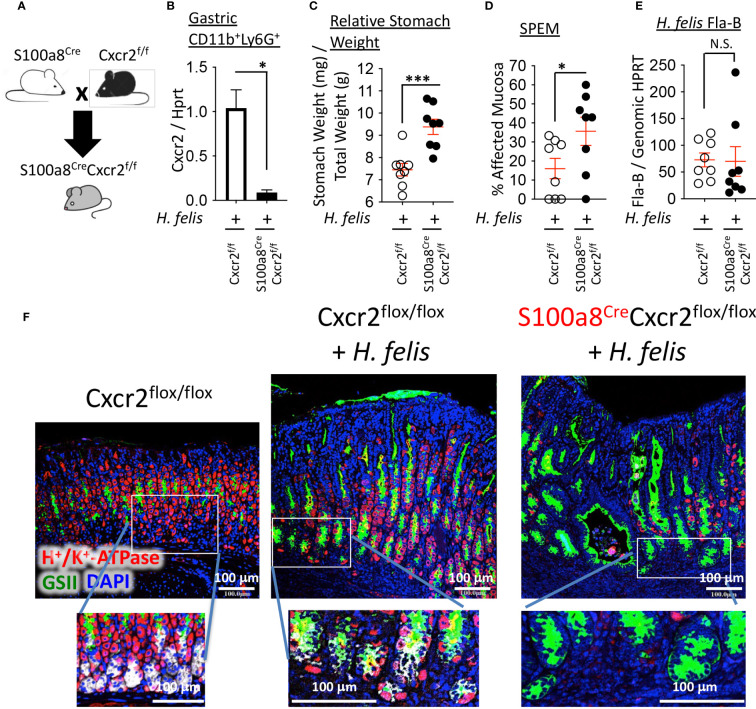
Ablation of Cxcr2 in S100a8-expressing cells exacerbates SPEM development. **(A)** Breeding scheme of S100a8^Cre^ mice with Cxcr2^flox/flox^ mice to generate S100a8^Cre^Cxcr2^flox/flox^ mice. **(B)** RT-qPCR analyses of Cxcr2 expression in FACS-isolated gastric CD11b^+^Ly6G^+^ cells from 6-month *H felis*-infected S100a8^Cre^Cxcr2^f/f^ versus Cxcr2^f/f^ stomachs. **(C)** Relative stomach weight in milligrams, normalized to total stomach weight in grams, in S100a8^Cre^Cxcr2^flox/flox^ mice versus Cxcr2^flox/flox^ controls after 6mo *H felis* infection. **(D)** Percentage area of SPEM in the gastric mucosae of 6mo *H felis*-infected S100a8^Cre^Cxcr2^flox/flox^ versus Cxcr2^flox/flox^ controls. SPEM quantification was performed using 4 longitudinal gastric strips, from the fundus and corpus, two of which were from the lesser and two from the greater curvatures of the stomach, and then by quantifying the percentage of 10x focal planes containing immunofluorescent co-localization of GSII and intrinsic factor, or expansion of GSII and total loss of intrinsic factor-expressing cells. **(E)** qPCR quantification of *H felis* flagellar filament B DNA, relative to genomic HPRT DNA, in 6mo *H felis*-infected S100a8^Cre^Cxcr2^flox/flox^ versus Cxcr2^flox/flox^ controls. **(F)** Representative immunofluorescent staining of H^+^/K^+^-ATPase (red), GSII (green), and DAPI (blue) in the upper panels, in addition to intrinsic factor (IF, white) in the lower inset panels, in 6mo *H felis*-infected S100a8^Cre^Cxcr2^flox/flox^ versus Cxcr2^flox/flox^ relative to uninfected control. Each data point represents one mouse. Error bars = means +/- SEM. *P < 0.05; ***P < 0.001.

To deplete Cxcr2 expression from S100a8^+^ myeloid cells, we crossed S100a8^Cre/+^ mice to Cxcr2^flox/flox^ mice on a C57BL/6 background (**Figure 5A**). This cross reduced Cxcr2 RNA expression in CD11b^+^Ly6G^+^ myeloid cells from 6mo *H. felis*-infected S100a8^Cre/+^Cxcr2^flox/flox^ mice relative to infected Cxcr2^flox/flox^ controls (**Figure 5B**). We then compared the gastric pathology following 6mo *H. felis* infection of S100a8^Cre/+^Cxcr2^flox/flox^ mice relative to infected Cxcr2^flox/flox^ controls. We observed that Cre/Flox-mediated ablation of Cxcr2 from S100a8^+^ G-MDSCs significantly increased gastric pathological parameters following 6mo *H. felis* infection, which included stomach weight relative to total mouse weight (**Figure 5C**) and the percentage area of SPEM, identified by either intrinsic factor and GSII co-localization and expansion of trans-differentiated mucous cells, and/or entire loss of chief cells and total gland expansion of GSII^+^ cells within the infected gastric mucosa (**Figures 5D, F**). Pathological exacerbation in the infected S100a8^Cre/+^Cxcr2^flox/flox^ stomachs, relative to infected Cxcr2^flox/flox^ stomachs, was confirmed by the Periodic acid Schiff (PAS)/alcian blue (AB) stain (**Supplementary Figure 13**). These differences were not attributed to a change in *H. felis* colonization as measured by *H. felis* flagellar filament B (Fla-B) DNA abundance (**Figure 5E**). The gastric pathological severity also appeared more pronounced in S100a8^Cre/+^Cxcr2^flox/flox^ mouse stomachs as marked by their total loss of intrinsic factor-expressing chief cells (grey staining in **Figure 5F**), and more dramatic parietal cell loss (red staining in **Figure 5F**), relative to Cxcr2^flox/flox^, which sustained the presence of some of their chief cells and parietal cells.

### S100a8^+^ cell-specific Cxcr2 ablation does not alter gastric immune cell frequency

As Cxcr2 has previously been shown to regulate G-MDSC infiltration in lower GI/colitis disease models (21), we assessed the frequency of gastric G-MDSC and non-G-MDSC myeloid cells, in S100a8^Cre/+^Cxcr2^flox/flox^ versus Cxcr2^flox/flox^ mice, by FACS. We did not observe differences in the frequency of gastric G-MDSC or non-G-MDSC myeloid cells between 6mo *H. felis*-infected S100a8^Cre/+^Cxcr2^flox/flox^ versus Cxcr2^flox/flox^ mice (**Supplementary Figures 14A–C**). We also did not observe differences in the frequency of other gastric immune cells, between 6mo *H. felis*-infected S100a8^Cre/+^Cxcr2^flox/flox^ versus Cxcr2^flox/flox^ mice, including CD4^+^ or CD8^+^ T cells (**Supplementary Figures 14D–F**), natural killer (NK) cells (**Supplementary Figures 14G, H**), or immature, mature naïve, or class-switched B cells (**Supplementary Figures 14I–L**). We conclude that Cxcr2 ablation within G-MDSCs does not affect gastric G-MDSC frequency, or the frequency of gastric non-G-MDSC myeloid cells, T cells, NK cells, or B cells.

### S100a8^+^ cell-specific Cxcr2 ablation associates with altered expression of lipid metabolism-associated genes in G-MDSCs and reduced Fabp5 expression

**Figure 6:**
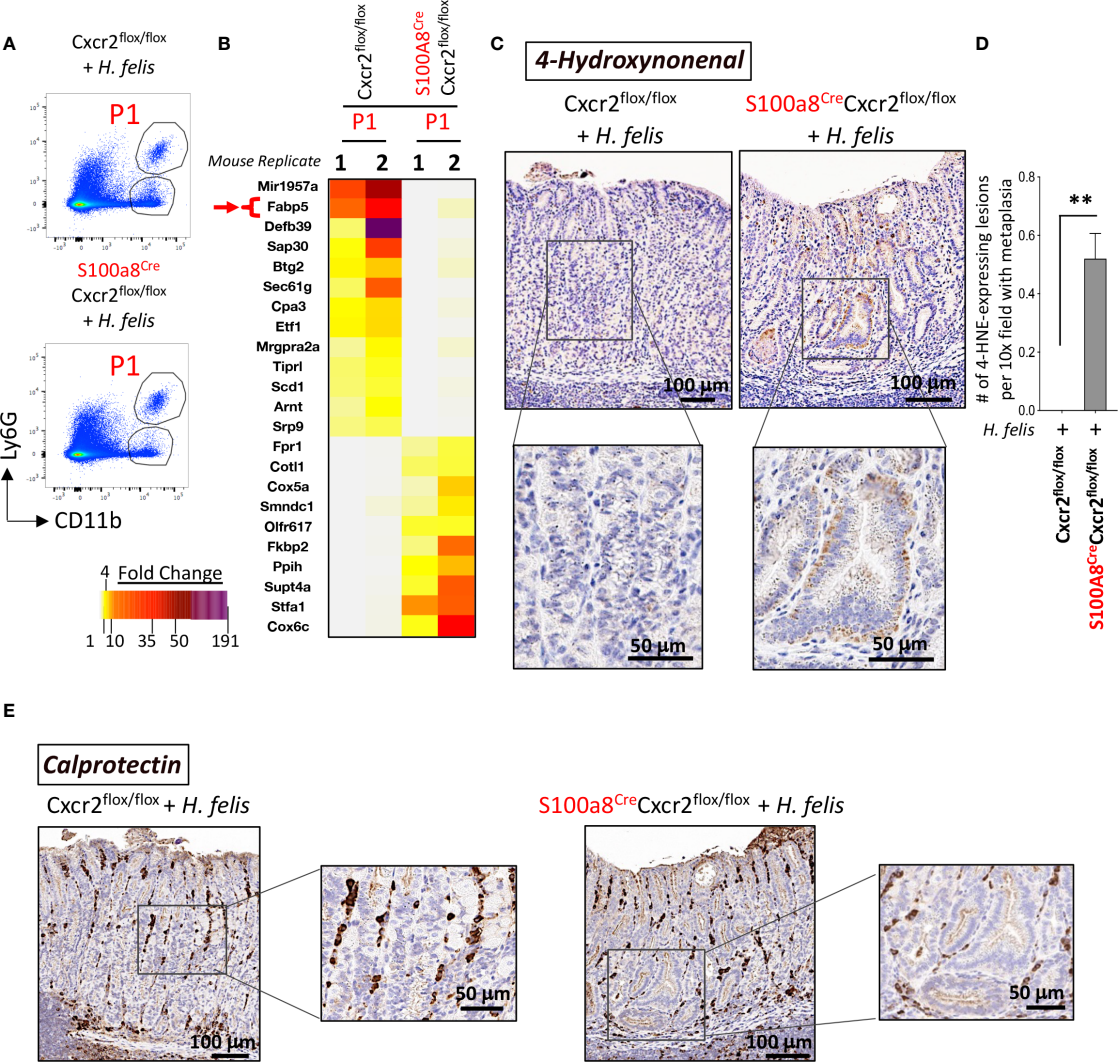
Cxcr2-deficient S100a8-expressing cells exhibit an altered expression of several genes associated with lipid metabolism, and an increase in the production of the lipid peroxidation product, 4-hydroxynonenal (4-HNE), within gastric epithelial lesions, following 6mo *H felis* infection. **(A)** Representative FACS plots of CD11b^+^Ly6G^+^ G-MDSCs from *H felis*-infected S100a8^Cre/+^Cxcr2^flox/flox^ versus Cxcr2^flox/flox^ stomachs, which were used for sorting gastric G-MDSCs for microarray analyses. **(B)** Microarray heatmap of the MDSC-specific genes that exhibited altered expression patterns in G-MDSCs in response to Cre/Flox-mediated Cxcr2 ablation. Fabp5 is directly involved in lipid peroxidation and is annotated by a red arrow. **(C)** Immunohistochemical staining of 4-HNE staining (brown), within gastric epithelial lesions, in 6mo *H felis*-infected S100a8^Cre/+^Cxcr2^flox/flox^ versus Cxcr2^flox/flox^ stomachs. **(D)** Quantitative morphometry of 4-HNE staining. **(E)** Representative immunohistochemical staining of calprotectin (brown) in 6mo *H felis*-infected S100a8^Cre/+^Cxcr2^flox/flox^ versus Cxcr2^flox/flox^ stomachs. **P < 0.01.

As Cxcr2 ablation in G-MDSCs did not modify gastric MDSC frequency, indicating a lack of effect on infiltration, we sought to determine the role of Cxcr2 in gastric G-MDSC function, and how it might regulate the severity of gastric pathology. We, therefore, performed FACS followed by a microarray of isolated gastric G-MDSCs from 6mo *H. felis*-infected S100a8^Cre/+^Cxcr2^flox/flox^ versus Cxcr2^flox/flox^ stomachs. Surprisingly, we found that Cxcr2 deficiency in G-MDSCs altered the expression of several genes associated with lipid metabolism such as the reduction in Fabp5, Sin3A Associated Protein 30 (Sap30), and B-cell translocation gene 2 (Btg2), and the upregulation of Coactosin-like protein (Cotl1), cytochrome c oxidase subunit 5a (Cox5a), and cytochrome c oxidase subunit 6c (Cox6c) (**Figures 6A, B**).

With regards to the downregulated genes, Fabp5 has been shown to function as an antioxidant protein, which scavenges reactive lipids thereby reducing lipid peroxidation (22). Sap30 and Btg2 are transcriptional targets of the retinoic acid receptor (RAR) signaling (23), which is known to reduce lipid peroxidation (24). Interestingly, Fabp5 can also bind and transport retinoic acid (RA) in the aqueous intracellular milieu (25, 26). Collectively, the downregulation of Fabp5, Sap30 and Btg2 indicated a disruption of RA signaling in Cxcr2-deficient MDSCs, which is known to regulate lipid peroxidation (24).

In contrast to the downregulated genes, we observed an upregulation of genes associated with an increase in lipid peroxidation such as Cotl1, which has been shown to interact with and stabilize 5-lipoxygenase (27, 28), the latter of which catalyzes the peroxidation of polyunsaturated fatty acids (29, 30). In addition, cytochrome c oxidase subunits Cox5a and Cox6c, whose dysregulation has been associated with altered production of reactive oxygen species (ROS) (31), which can in turn induce lipid peroxidation (32), were also upregulated.

Given the observed downregulation of genes whose products restrict lipid peroxidation, and upregulation of those which induce lipid peroxidation, we sought to determine the effect of MDSC Cxcr2 deficiency on lipid peroxidation in the infected gastric mucosa.

### S100a8^Cre/+^Cxcr2^flox/flox^ mice exhibit lower lipid peroxidation in the gastric mucosa during *H. felis* infection

**Figure 7:**
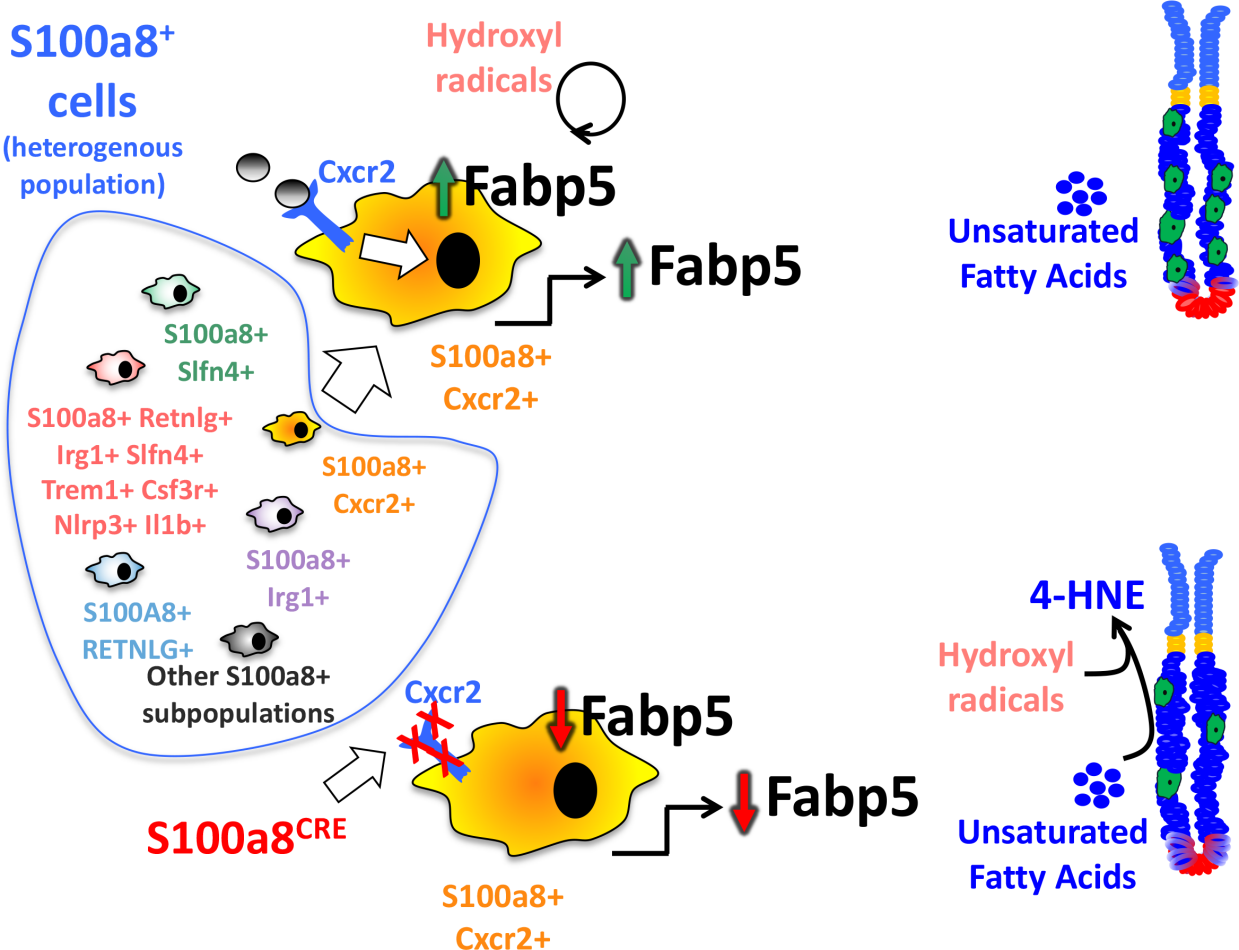
Hypothetical modeling of the role of Cxcr2 within S100a8-expressing G-MDSCs. S100a8 is a pan marker that comprises heterogeneous subsets of cells expressing our identified markers. These subsets are non-distinct and overlapping. This diagram also represents one specific population that expresses all our identified markers concomitantly at higher levels than the rest of the S100a8+ populations. The absence of Cxcr2 in S100a8^Cre^Cxcr2^floxflox^ G-MDSCs alters the expression of genes involved in lipid metabolism, among which is a reduction of Fabp5, which plays a direct role in regulating lipid peroxidation by scavenging hydroxyl radicals. The downregulation of Fabp5, therefore, leads to increased lipid peroxidation within epithelial cells, and the production of lipid peroxidation product 4-HNE, which exacerbates gastric epithelial lesions.

Fabp5 has previously been shown to suppress lipid peroxidation in viral-induced lung inflammation (33). Overall, fatty acid binding proteins (FABPs) are known to scavenge hydroxyl radicals involved in the lipid peroxidation (34). MDSC are known to regulate lipid metabolism in the inflammatory microenvironment, and express several transport receptors involved in lipid uptake and scavenging (35), including CD36, macrophage scavenger receptor 1 (Msr1)/CD204, and fatty acid transporters 1 and 6 (Fatp1 and 6) (36). Since MDSCs are usually involved in lipid uptake, and since they lost their expression of FABP5 in the chronically inflamed S100a8^Cre^Cxcr2^f/f^ stomachs, which normally scavenges hydroxyl radicals, and since the increase in hydroxyl radicals can trigger DNA damage in epithelial cells (37), we postulated that the reduction in Fabp5 in S100a8^Cre^Cxcr2^f/f^ G-MDSCs leads to the accumulation of hydroxyl radicals within the gastric microenvironment and higher lipid peroxidation by epithelial cells. One of the final products of lipid peroxidation is 4-Hydroxynonenal (4-HNE). We, therefore, stained tissue sections from 6mo *H. felis*-infected S100a8^Cre/+^Cxcr2^flox/flox^ versus Cxcr2^flox/flox^ mouse stomachs with 4-HNE antibody to firstly detect the areas in which lipid peroxidation was occurring (**Figure 6C**), and secondly with calprotectin to identify the location in which MDSCs might be scavenging hydroxyl radicals *via* FABP5 activity within the gastric mucosa (**Figure 6E**). We observed a significant increase in 4-HNE production signifying increased lipid peroxidation in the gastric mucosa of 6mo *H. felis*-infected S100a8^Cre/+^Cxcr2^flox/flox^ relative to Cxcr2^flox/flox^ mice (**Figures 6C, D**). The localization of G-MDSCs marked by calprotectin staining appeared to span the stroma adjacent to epithelial lesions (**Figure 6E**). We posit that Cxcr2 deficiency in MDSCs reduces Fabp5 expression in these cells, thereby reducing scavenging of hydroxyl radicals within the gastric microenvironment, and increasing the ensuing lipid peroxidation within gastric epithelial cells, which is marked by 4-HNE production. A hypothetical model for the function of MDSC Cxcr2 in gastric pathology is presented in **Figure 7**.

### Characterization of the overlap versus distinctness of the gastric G-MDSC/non-G-MDSC classification relative to the gastric F4/80^+^ macrophage and CD11c^+^ dendritic cell classifications

Following the characterization of the effect of Cxcr2 on gastric G-MDSCs in gastric pathology, it remained unclear what this myeloid cell classification (i.e., CD11b^+^Ly6G^+^/S100a8^+^ for G-MDSCs) represented relative to the gut macrophage marker classification utilizing F4/80^+^ (38) or the traditional gut dendritic cell classification utilizing CD103^+^CD11c^+^ (39). We therefore characterized the overlap versus distinctness between these cells relative to each marker classification.

We observed that gastric CD11b^+^Ly6G^+^ G-MDSCs represented a 68% overlap with F4/80^+^ gastric myeloid cells, and a 1% overlap with myeloid cells that co-expressed both gut macrophage (F4/80^+^) and dendritic cell (CD103^+^CD11c^+^) classification markers, while 29% of G-MDSC cells neither expressed macrophage nor dendritic cell classification markers (F4/80^-^CD103^-^CD11c^-^) (**Supplementary Figures 15–17**). Non-G-MDSC gastric myeloid cells (CD11b^+^Ly6G^-^) consisted of F4/80^+^ myeloid cells (71%) and monocytic MDSCs (M-MDSCs) (29%) (**Supplementary Figures 15–17**).

We therefore conclude that the gastric myeloid cell classifications are not distinct, and represent significant overlap with other classifications regardless of which classification is being used. In this paper, we provide one avenue for the phenotypic classification of gastric myeloid cells by the G-MDSC versus non-G-MDSC classification using the pan-marker, S100a8, which enables functional investigation of the heterogeneous myeloid cell subsets of this specific population.

## Discussion

This study characterizes the heterogeneity of gastric G-MDSCs and identifies calprotectin expression as a pan marker for these cells. A Cxcr2-expressing cellular subset of this gastric G-MDSC population regulates *Helicobacter*-mediated immunopathological outcome, and is associated with the regulation of lipid peroxidation. The study first identifies calprotectin (S100a8 and S100a9 subunits) as a pan-specific marker of gastric G-MDSCs and further uncovers the expression profile of these gastric G-MDSCs. The high expression of the previously identified marker of gastric G-MDSCs, Slfn4 (2, 5), in our microarray analyses of CD11b^+^Ly6G^+^ gastric MDSCs, and our scRNA-Seq of S100a8^+^ MDSCs, further validated our characterization of these cells. Multiple highly enriched genes within the gastric G-MDSC population do not represent distinct, but rather overlapping, sub-populations of the total S100a8^+^ G-MDSC population, therefore corroborating its heterogeneity. Elucidating the functions of each of these enriched marker genes within this heterogeneous population can help characterize this population’s heterogeneous functions.

As we observed high expression of Cxcr2 in gastric S100a8^+^ G-MDSCs, we utilized the S100a8-Cre driver to ablate Cxcr2 expression from these cells. The outcome was that this ablation did not affect myeloid or immune cell frequency, but was rather associated with enhanced lipid peroxidation mediated by the gastric MDSC population, which exacerbated gastric immunopathology. We conclude that the Cxcr2-expressing gastric G-MDSC population regulates gastric mucosal damage in a manner that is associated with dysregulated lipid peroxidation

Gastric G-MDSCs were first identified in one study in which gastric-specific overexpression of IL-1β induced the appearance of these G-MDSCs within gastric tumors that developed in this mouse model (1). Our group later identified the expression of a specific marker, called SLFN4, in a subset of gastric myeloid cells induced by chronic *Helicobacter felis* infection of the stomach (5), which were later identified to be G-MDSCs (2). Therefore, the appearance of the G-MDSC population in the gastric mucosa does not only pertain to the tumor setting but also occurs during chronic gastric inflammation, which associates with the development of pre-neoplastic lesions. The Slfn4^+^ G-MDSCs, during chronic *Helicobacter* infection, were later validated to possess the ability to suppress T cell activity (2). In the gastric tumor setting, reduction of MDSC accumulation in mouse models by the treatment with 5-fluorouracil and oxaliplatin increased the effects of anti-programmed cell death protein 1 (anti-PD-1) treatment, which promotes CD8^+^ T cell tumor infiltration (40). In human gastric cancers, MDSCs are increased in circulation (41) and cancer tissue (42), and they significantly correlate with plasma levels of calprotectin (S100a8/9) (41), as well as with lower survival rates (41, 42). Therefore, the overarching conclusion about the function of gastric MDSCs in tumors resides in their ability to suppress anti-tumor immunity by suppressing cytotoxic T cell activity. However, their role in gastric preneoplastic pathology remains unclear, especially when increases in CD8^+^ T cells during chronic gastric inflammation have been associated with worsened pathology (43). In this study, we show that G-MDSCs can play distinct functions from T cell suppression, suggesting that their previously described functions in promoting gastric immunopathology (38, 44, 45) or otherwise suppressing such immunopathology (1, 2, 40) require further careful investigation and that concomitant functions might operate within these same cells to enhance and reduce pathology simultaneously. Therefore, in this study, we are not claiming that the novel function we observed to be associated with dysregulated lipid peroxidation is the sole function of G-MDSCs, but perhaps one of several functions and/or responses that these cells can elicit within the chronically inflamed gastric microenvironment. The significance of our findings, however, is to provide an avenue and a proof-of-concept study that demonstrates the ability to isolate and decipher distinct functions that can be modulated by different pathways within gastric G-MDSCs. We identify the markers expressed by gastric G-MDSCs, as well as an example of how one promoter can be utilized to specifically target one of their specific cellular pathways.

We observed in this study that Cxcr2-deficiency in G-MDSCs was associated with enhanced lipid peroxidation, which is in line with previous literature documenting the importance of fatty acid oxidation in regulating MDSC function [as reviewed in (46, 47)]. Indeed, lipid uptake by MDSCs has been shown to regulate their function in the tumor microenvironment and their ability to suppress T cell activity (48). We did not observe any evidence of modified gastric T cell frequencies in this study. However, we did observe a reduction in Fabp5 in Cxcr2-deficient gastric G-MDSCs. Fatty acid-binding proteins (FABPs) are generally involved in the trafficking of lipids (49), and Fabp5 in particular has been shown to be induced in tumor-infiltrating MDSCs relative to splenic MDSCs and immature myeloid cells (36), indicating its induction in MDSCs within inflammatory environments. Since Fabp5 suppresses lipid peroxidation and oxidative damage in the inflammatory environment (33) and possesses the ability to scavenge reactive lipids such as 4-HNE (22), we posit that the reduction of Fabp5 in Cxcr2-deficient MDSCs increases lipid peroxidation, which can in turn increase pathology by increasing DNA damage in epithelial cells [the effect of lipid peroxidation on DNA damage has been previously reviewed in (37)].

Confirmatory to our hypothesis, the exacerbation of lipid peroxidation in the Cxcr2-deficient G-MDSCs did not only associate with the reduction of Fabp5 expression, but also with other changes associated with lipid peroxidation including (i) a reduction in transcriptional targets of retinoic acid receptor (RAR) signaling (Sap30 and Btg2) (23), which are known to reduce lipid peroxidation (24), (ii) an upregulation of Cotl1, which has been shown to interact with and stabilize 5-lipoxygenase (27, 28), which in turn catalyzes the peroxidation of polyunsaturated fatty acids (29, 30), and (iii) an upregulation of Cox5a and Cox6c, which regulate ROS production (31) that can, in turn, induce lipid peroxidation (32). Hence the overall changes in Cxcr2-deficient G-MDSCs indicate an association with increased in lipid peroxidation, which was confirmed by the assessment of 4-HNE levels in the gastric tissue.

Even though our data supports an association with dysregulated lipid peroxidation in response to Cxcr2 deficiency in G-MDSCs, the mechanism in which Cxcr2 deficiency within G-MDSCs might regulate lipid peroxidation remains unclear. Our future studies will aim to determine the role of Cxcr2 in G-MDSC function pertaining to lipid metabolism and oxidative damage. Another limitation of our study is that the S100a8^Cre^ driver is expected to target Cxcr2 in circulating neutrophils, and not only in gastric G-MDSCs. However, G-MDSCs are arguably regarded as long-lived neutrophils (7), and targeting G-MDSCs independently of circulating neutrophils is not possible. Moreover, the S100a8^Cre^ driver that we used was not inducible, and which is therefore expected to concomitantly lead to the ablation of Cxcr2 expression in a 10% to 20% fraction of granulocyte-monocyte progenitor (GMP) cells (50), and not only gastric G-MDSC cells. However, the observation that the 6mo *H. felis*-infected S100a8^Cre^Cxcr2^fl/fl^ mice did not exhibit altered frequencies in myeloid cells of the gastric mucosa, and the specificity of high S100a8 expression within the gastric G-MDSC population of the stomach, favors the hypothesis that our observed effects are due to the dysregulation of G-MDSC function in the chronically inflamed stomach S100a8^Cre^Cxcr2^fl/fl^. Follow-up studies utilizing an inducible S100a8^Cre^ mouse model are necessary to investigate the function of this promoter as a gastric G-MDSC pan-specific marker.

We conclude, based on the evidence in this study, that G-MDSC Cxcr2 regulates gastric immunopathology, which can at least in part be attributed to an association with dysregulated lipid peroxidation in the absence of Cxcr2.

## Data availability statement

The data presented in this study are deposited in NCBI's Gene Expression Omnibus and are accessible for (1) scRNA-Seq data through GEO series accession number GSE240709, and for (2) the microarray data through GEO series accession number GSE240720.

## Ethics statement

The animal study was approved by Committee on Use and Care of Animals at the University of Michigan under protocol #PRO00009914. The study was conducted in accordance with the local legislation and institutional requirements.

## Author contributions

ME-Z designed the research, HG contributed to intellectual content, and KK and ME-Z performed the experiments. All authors contributed to the article and approved the submitted version.
