# Supplementary material for: The Cxcr2+ subset of the S100a8+ gastric granylocytic myeloid-derived suppressor cell population (G-MDSC) regulates gastric pathology
Source: Front Immunol. 2023 Sep 8;14:1147695. doi: 10.3389/fimmu.2023.1147695 (PMC10514515; doi:10.3389/fimmu.2023.1147695)
Supplement: Supplementary file 1 [file DataSheet_1.pdf]

Supplementary Figure 1

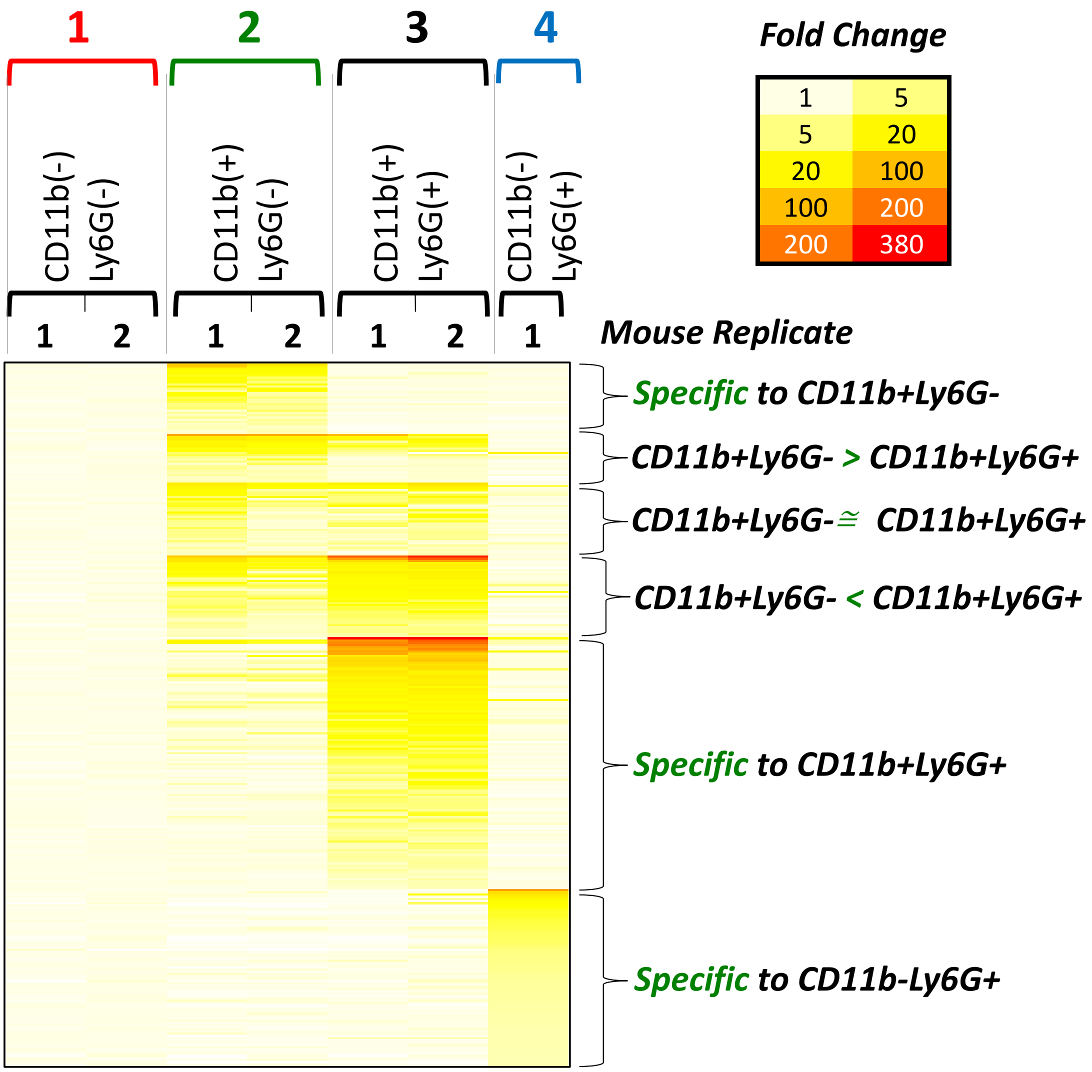

Supplementary Figure 1. Microarray heatmap profile comparison between gastric $CD11b^{-}Ly6G^{-}$ non-myeloid cells, gastric $CD11b^{+}Ly6G^{-}$ non-G-MDSC myeloid cells (annotated as green font "2"), gastric $CD11b^{+}Ly6G^{+}$ G-MDSC myeloid cells (annotated as black font "3"), and gastric $CD11b^{-}Ly6G^{+}$ non-myeloid cells (annotated as blue font "4"), based on the annotations in the FACS plots in **Figure 1A**. The heatmap was organized from top to bottom according to the expression levels of the genes in $CD11b^{+}Ly6G^{-}$, $CD11b^{+}Ly6G^{+}$, and $CD11b^{-}Ly6G^{+}$ as annotated on the right side of the heatmap. The heatmap for each cell population displayed was obtained from two mouse replicates (Mouse 1 and Mouse 2), except for the $CD11b^{-}Ly6G^{+}$ non-myeloid control population, which was only obtained from one mouse (Mouse 1). Fold change values are shown on the top right.

Supplementary Figure 2

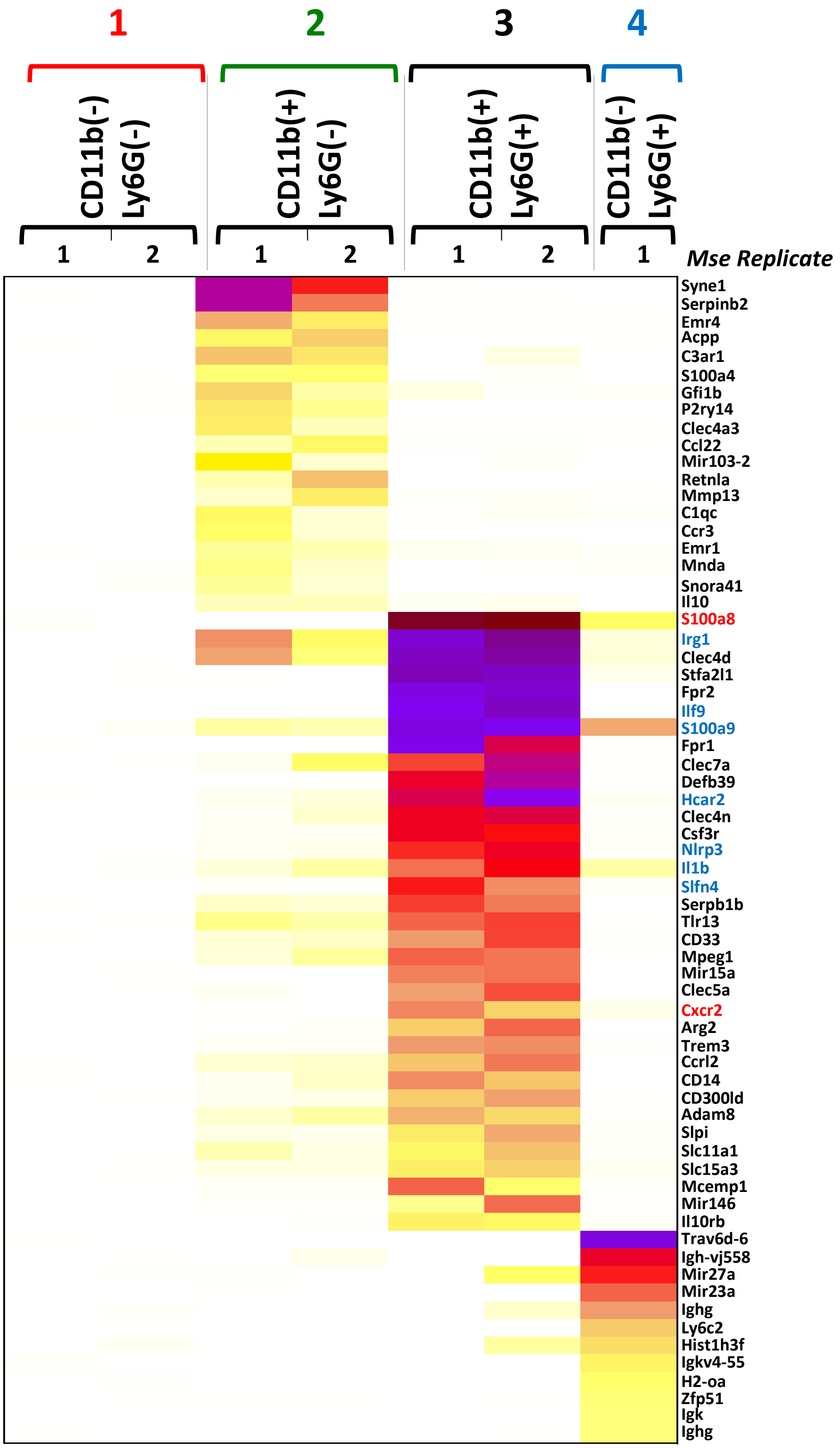

Supplementary Figure 2. Microarray heatmap annotating the gene list from **Figure 1B**, of the highest expressed genes that are specifically enriched in gastric CD11b^{+}Ly6G^{-} non-G-MDSC

myeloid cells (annotated as green font “2”), versus those specifically enriched in gastric $CD11b^+Ly6G^+$ G-MDSC myeloid cells (annotated as black font “3”), and those specifically enriched in non-meyloid gastric $CD11b^-Ly6G^+$ control cells (annotated as blue font “3”).

Supplementary Figure 3

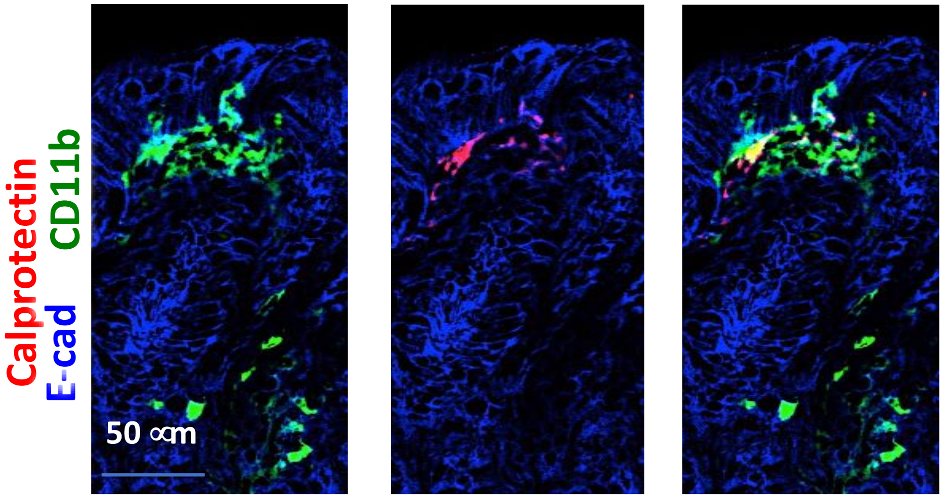

Supplementary Figure 3. Representative image of the immunofluorescent staining of CD11b (green), calprotectin (red), and E-cadherin (blue) in the 6mo *H. felis*-infected mouse gastric mucosa.

Supplementary Figure 4

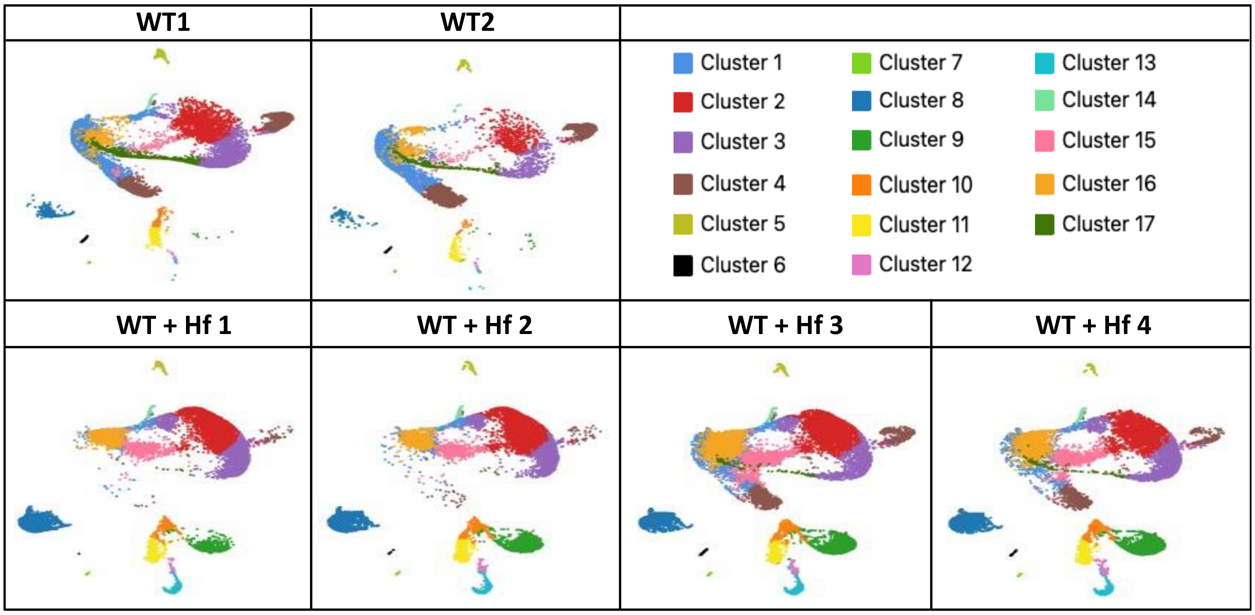

Supplementary Figure 4. ScRNA-Seq cell clustering by based on the 10x Genomics Loupe browser software. The genes enriched in each cluster are listed in Supplementary Table 1. The UMAP plots from the third uninfected and fifth *H. felis*-infected mice are not shown here because they were 3’ sequenced, and therefore cannot be aggregated with this data set, which was 5’ sequenced.

Supplementary Figure 5

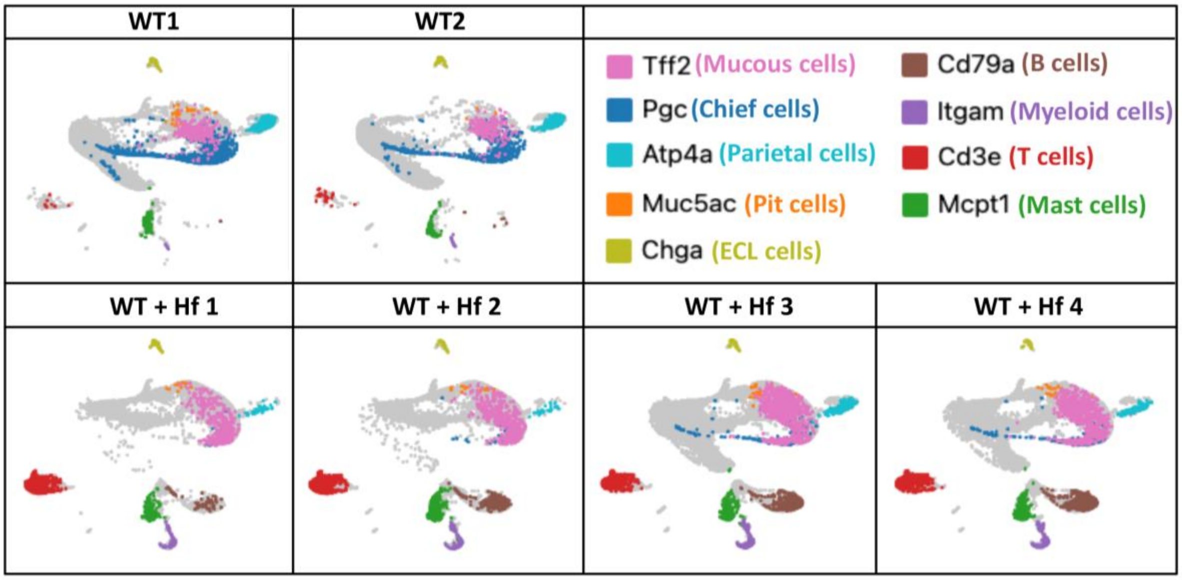

Supplementary Figure 5. Individual scRNA-Seq UMAP plots from two uninfected and four 6 month *H. felis*-infected stomachs, for the data shown in **Figures 3A, B**. The UMAP plots from the third uninfected and fifth *H. felis*-infected mice are not shown here because they were 3' sequenced, and therefore cannot be aggregated with this data set, which was 5' sequenced.

Supplementary Figure 6

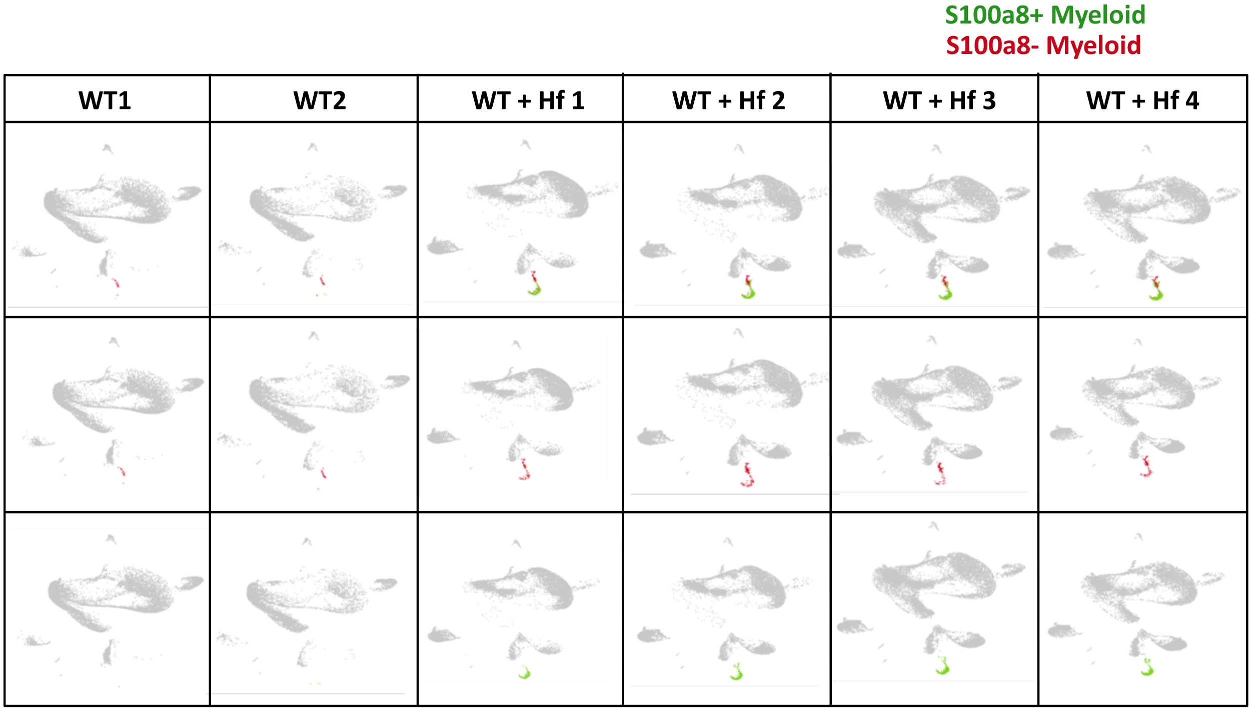

Supplementary Figure 6. Individual scRNA-Seq UMAP plots from two uninfected and four 6 month *H. felis*-infected stomachs, for the data shown in **Figures 3C, D**. The data shows the expansion of S100a8$^-$ and S100a8$^+$ myeloid cells in the gastric mucosae of individual mouse stomachs following 6 month *H. felis* infection. The UMAP plots from the third uninfected and fifth *H. felis*-infected mice are not shown here because they were 3' sequenced, and therefore cannot be aggregated with this data set, which was 5' sequenced. The *upper panels* show the S100a8$^-$ (red) and S100a8$^+$ (green) cells in the same plot. The *middle panels* show the S100a8$^-$ (red) but exclude the S100a8$^+$ (green) cells. The *lower panels* show the S100a8$^+$ (green) cells but exclude the S100a8$^-$ (red) cells.

Supplementary Figure 7

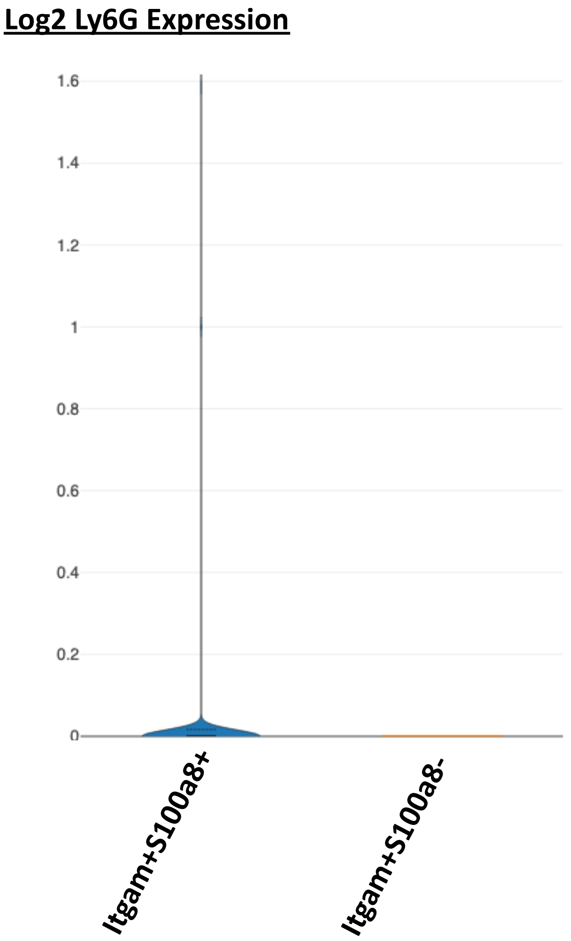

Supplementary Figure 7. Violin plots, pooled from scRNA-Seq data of 6 month *H. felis*-infected stomachs from 4 mice, showing the distribution of expression of Ly6G in $Itgam^+S100a8^+$ versus $Itgam^+S100a8^-$ myeloid cells. Itgam represents the CD11b gene.

Supplementary Figure 8

(A)

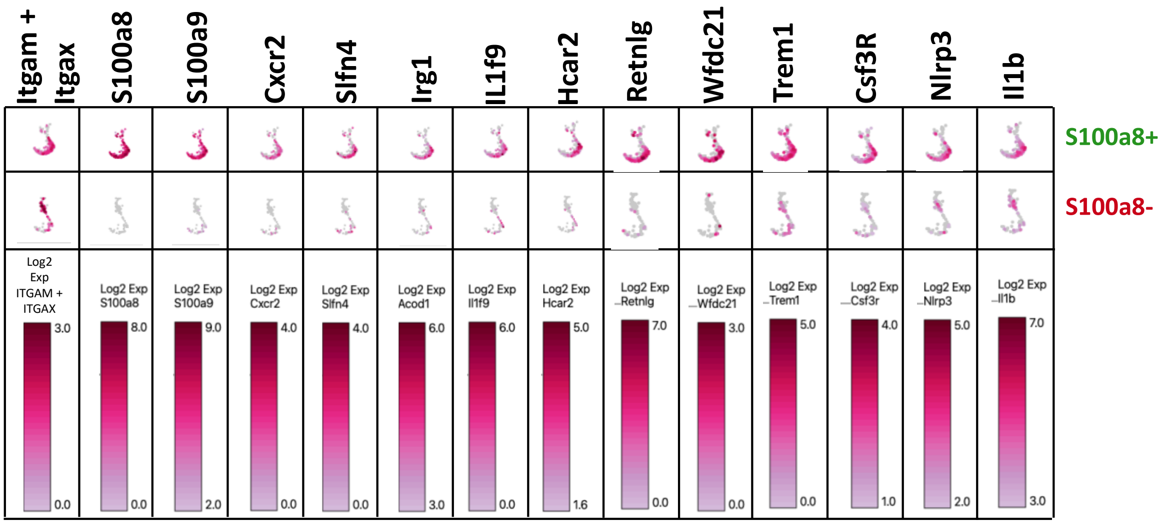

(B)

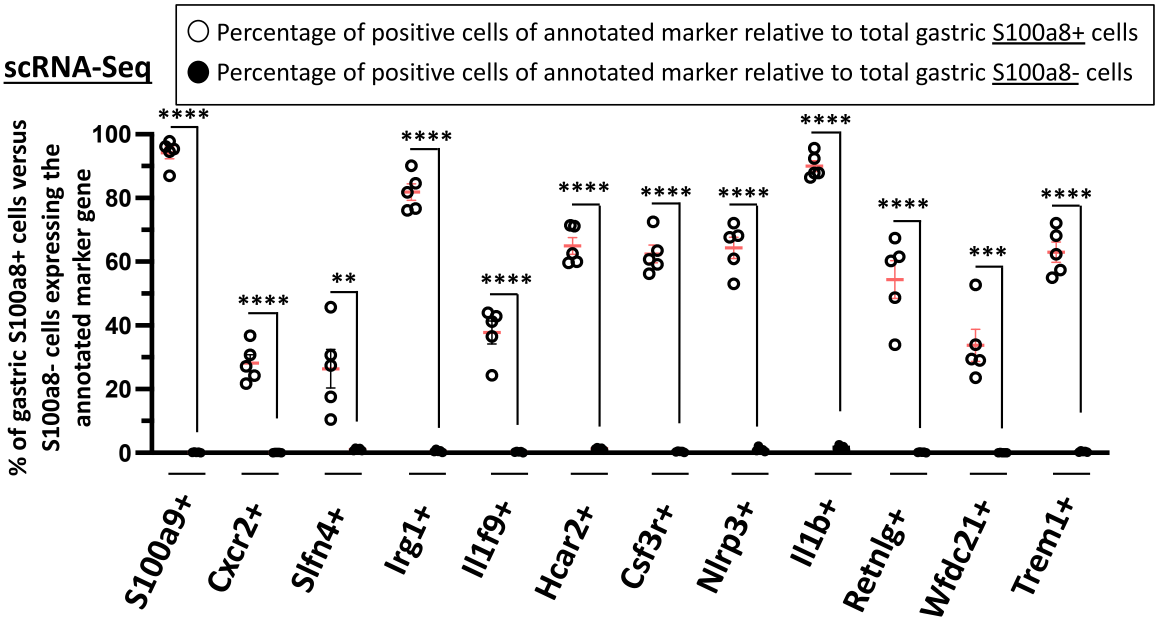

Supplementary Figure 8. (A) Expression levels of specific S100a8$^+$ G-MDSC markers S100a9, Cxcr2, Slfn4, Irg1, Il1f9, Hcar2, Csf3rR, Nlrp3, and Il1b within S100a8$^+$ versus S100a8$^-$ gastric myeloid cells, which were simultaneously identified by microarray in CD11b$^+$Ly6G$^+$ MDSCs in **Figure 1B**. Three additional markers, Retnlg, Wfdc21, and Trem1, which were not identified by microarray in **Figure 1B**, but detected by scRNA-Seq, are also shown. Fold change bars are shown below each marker. **(B)** Quantification by scRNA-Seq of the percentages of cells expressing the markers from (A), relative to total gastric S100a8$^+$ cells versus total gastric S100a8$^-$ cells. Each datapoint represents one mouse. Error bars = means +/- SEM. ****P < 0.0001; ***P < 0.001; **P < 0.01.

Supplementary Figure 9

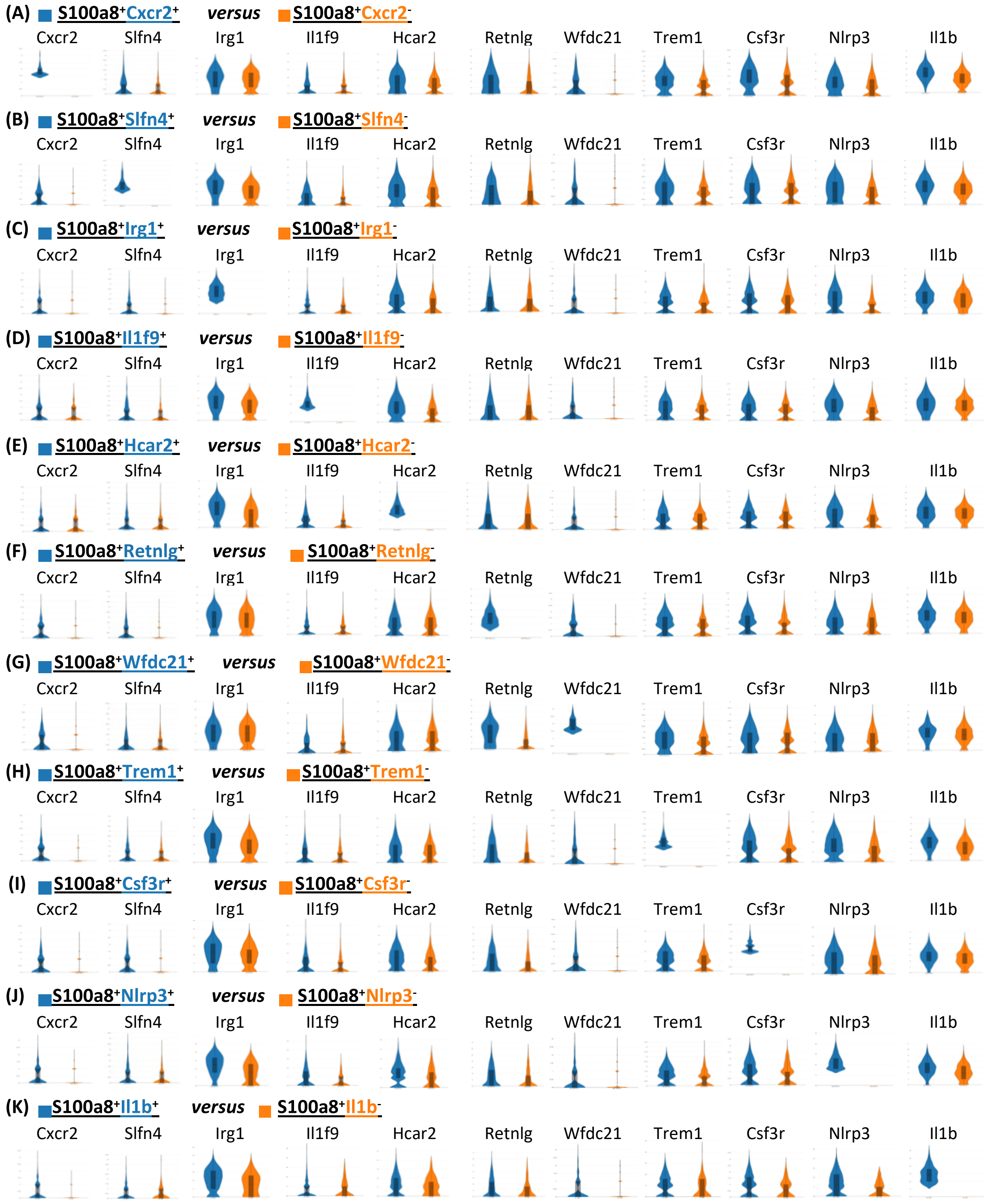

Supplementary Figure 9. Violin plots, pooled from scRNA-Seq data of 6 month *H. felis*-infected stomachs from 4 mice, outlining the relationships between S1008^{+} myeloid cell sub-populations expressing versus lacking each of our identified marker genes that are enriched within S1008^{+} myeloid cells.

Supplementary Figure 10

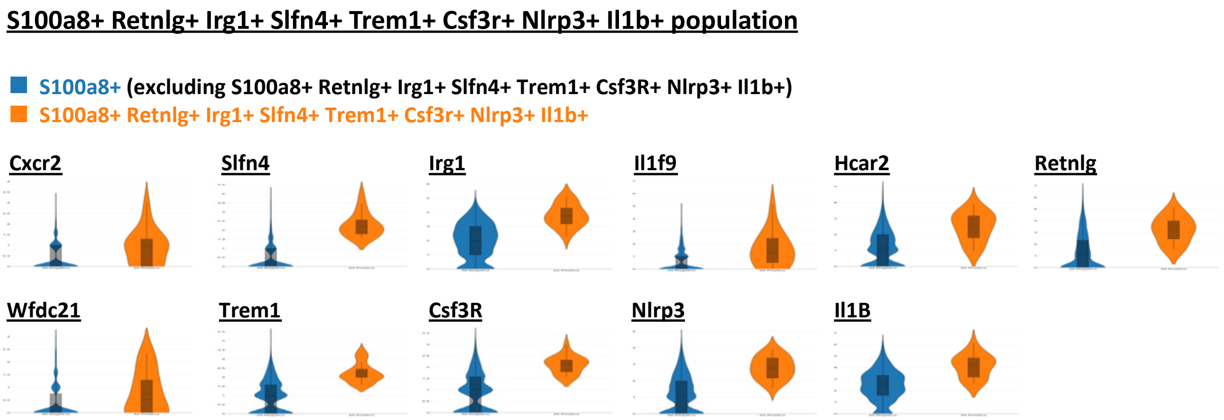

Supplementary Figure 10. Violin plots, pooled from scRNA-Seq data of 6 month *H. felis*-infected stomachs from 4 mice, outlining the expression pattern of our identified gastric G-MDSC markers within the gastric S100a8$^+$Retnlg$^+$Irg1$^+$Slfn4$^+$Trem1$^+$Csf3r$^+$Nlrp3$^+$Il1b$^+$ population relative to the remaning S100a8$^+$ gastric G-MDSC population.

Supplementary Figure 11

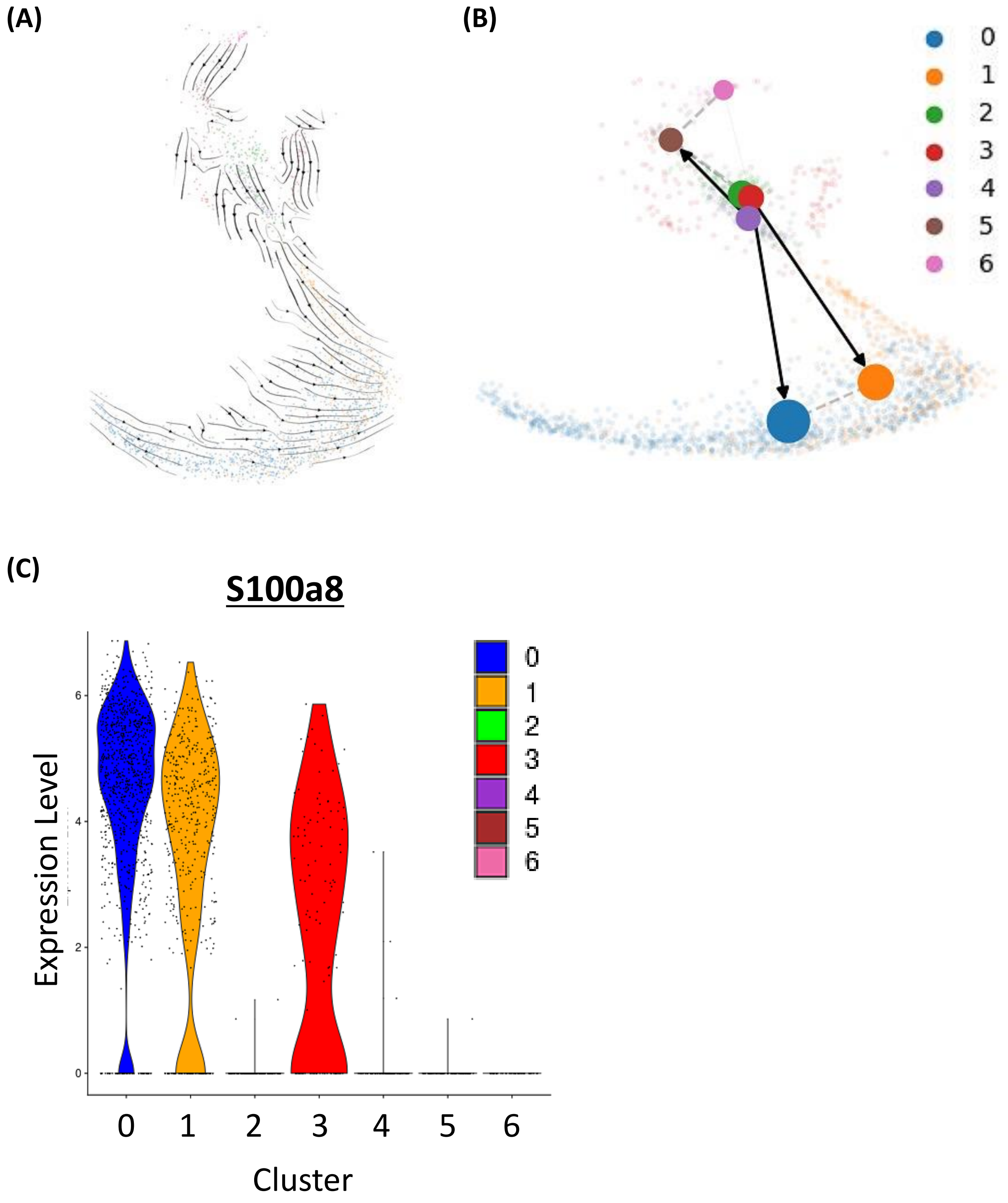

Supplementary Figure 11. Velocity and PAGA plots for the gastric myeloid cell subpopulation. **(A)** Velocity plot and **(B)** PAGA plot obtained from the myeloid cell subclusters pooled from 4 *H. felis*-infected stomachs. **(C)** Violin plots of S100a8 expression in the 7 myeloid cell subclusters obtained by unsupervised analyses.

Supplementary Figure 12

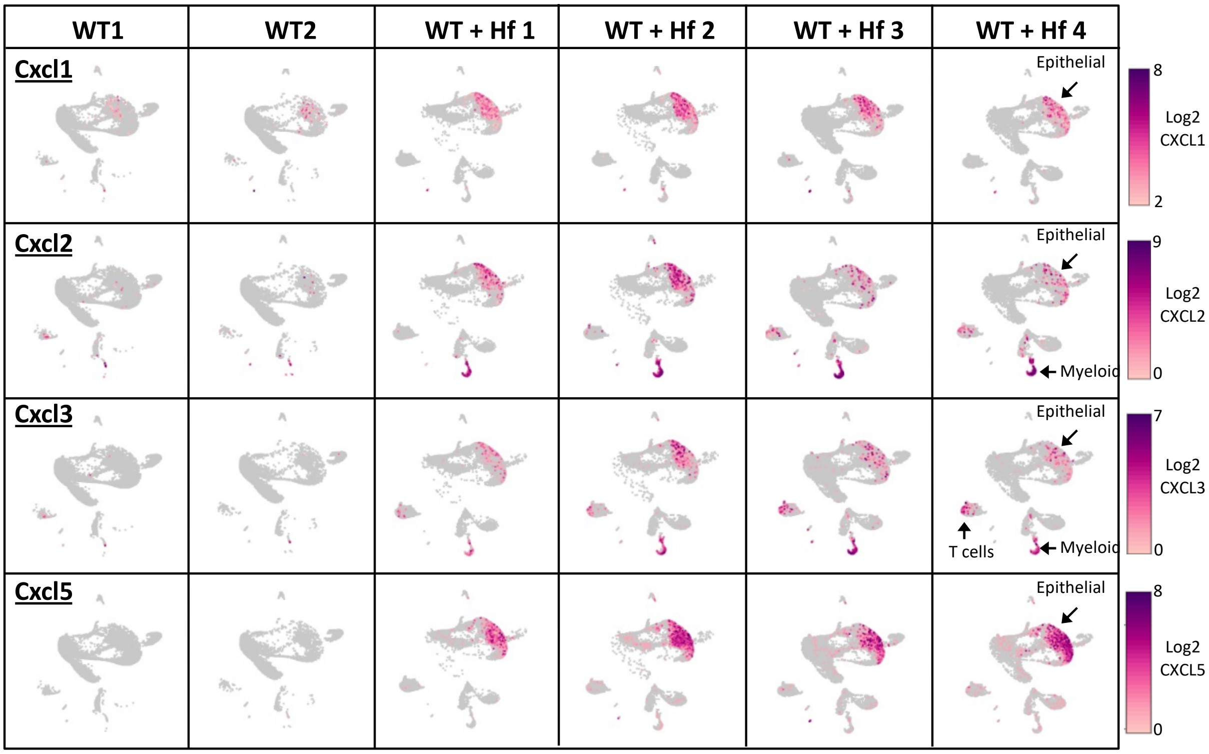

Supplementary Figure 12. ScRNA-Seq UMAP plots from 2 uninfected and 4 *H. felis*-infected mouse stomachs showing the induction of Cxcr2 ligand genes Cxcl1, Cxcl2, Cxcl3 and Cxcl5. Cxcl1 and Cxcl5 are induced in gastric epithelial cells following infection, and Cxcl2 and Cxcl3 are induced in gastric epithelial, myeloid and T cells as annotated.

Supplementary Figure 13

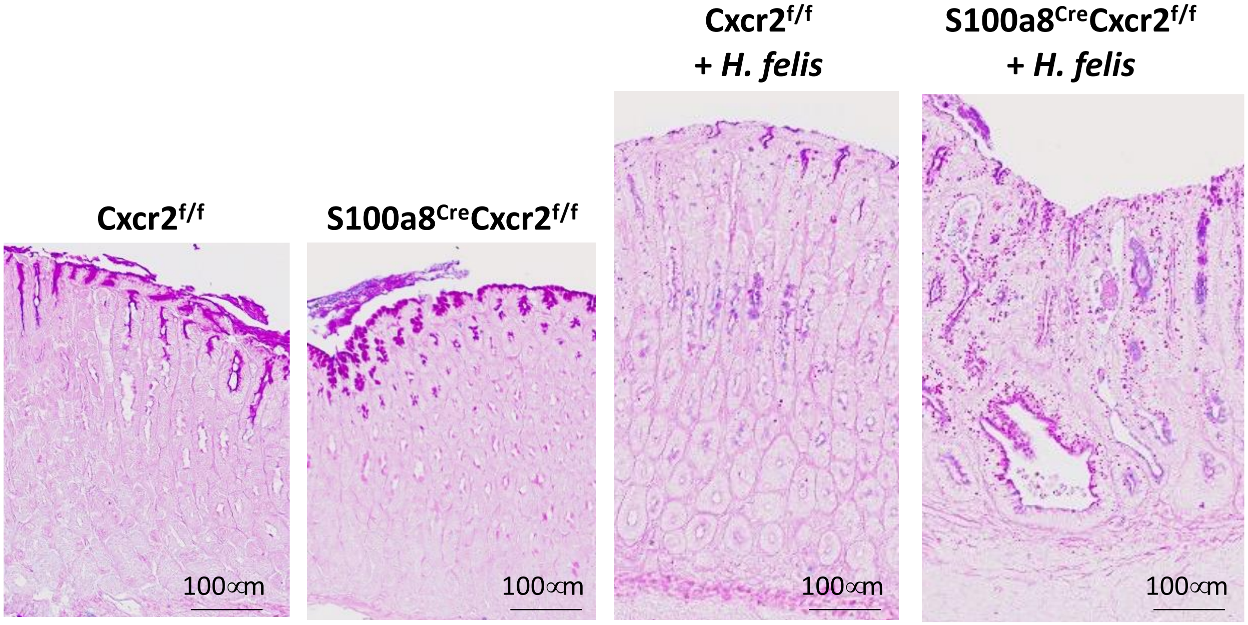

Supplementary Figure 13. Representative images showing the staining with Periodic acid-Schiff (PAS) and alcian blue (AB) in 6-month *H. felis*-infected S100a8^{Cre}Cxcr2$^{f/f}$ versus Cxcr2$^{f/f}$ stomachs relative to uinfected controls.

Supplementary Figure 14

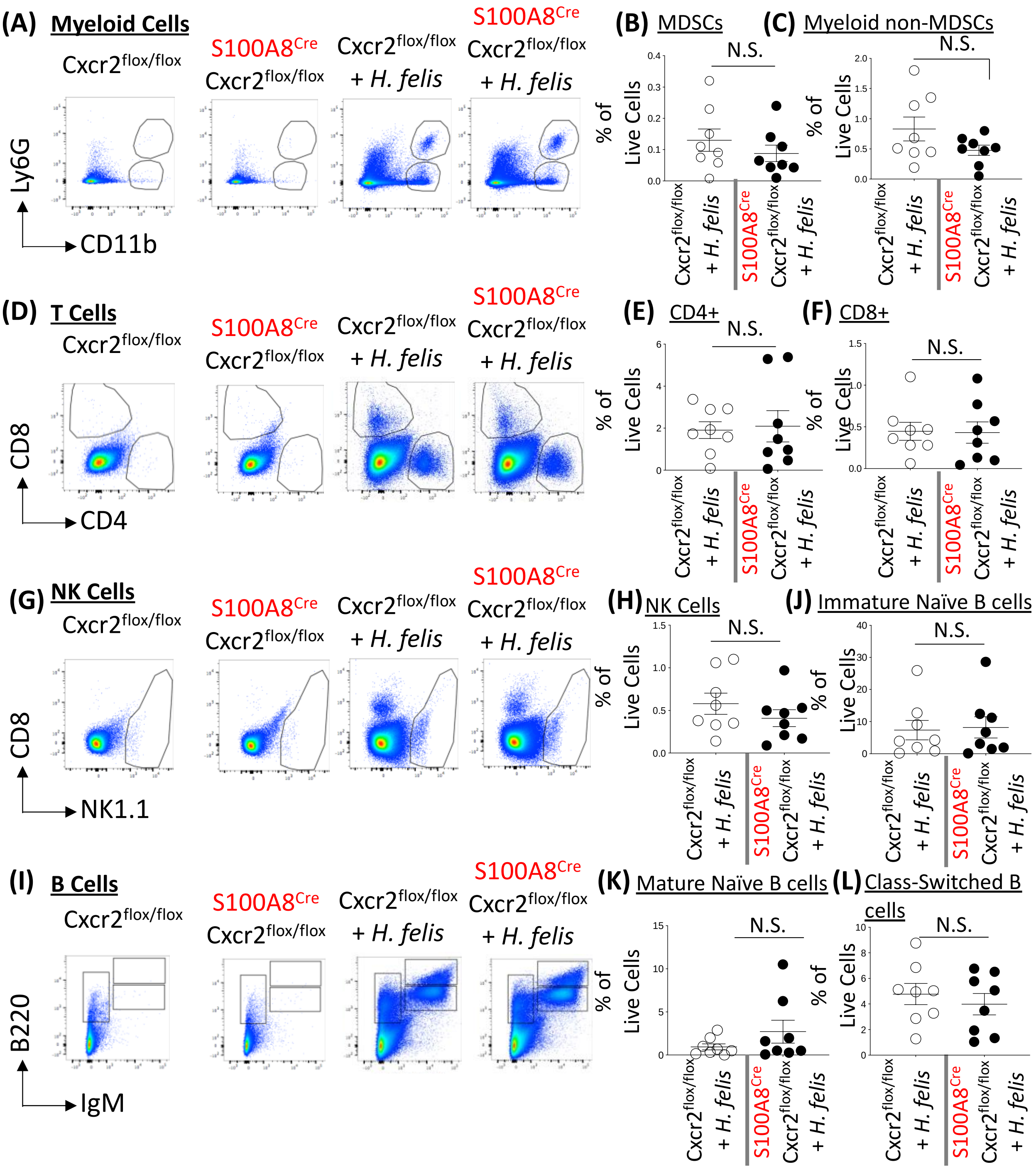

Supplementary Figure 14. Ablation of Cxcr2 in S100a8-expressing cells does not alter the frequency of gastric immune cells within the 6mo *H. felis*-infected gastric mucosa. (*A-C*) Representative FACS plots of gastric G-MDSC ($CD11b^+Ly6G^+$) versus non-G-MDSC ($CD11b^+Ly6G^-$) myeloid cells in 6mo *H. felis*-infected $S100a8^{Cre}Cxcr2^{flox/flox}$ stomachs versus $Cxcr2^{flox/flox}$ controls, relative to uninfected. Frequncies from 8 mice per infected group are shown in (*B*) and (*C*) for G-MDSC and non-G-MDSC myeloid cells respectively. (*D-F*) Representative

FACS plots of gastric CD4$^+$ and CD8$^+$ T cells in 6mo *H. felis*-infected S100a8^{Cre}Cxcr2$^{flox/flox}$ stomachs versus Cxcr2$^{flox/flox}$ controls, relative to uninfected. The frequencies from 8 mice per infected group are shown in (*E*) and (*F*). (*G-H*) Representative FACS plots of NK1.1$^+$ natural killer cells (*G*), with the frequencies from the infected groups shown in (*H*). (*I-L*) Representative FACS plots of IgM$^+$B220^{low} immature B cells, IgM$^+$B220$^+$ mature B cells, and IgM$^-$B220$^+$ class-switched B cells in 6mo *H. felis*-infected S100a8^{Cre}Cxcr2$^{flox/flox}$ stomachs versus Cxcr2$^{flox/flox}$ controls, relative to uninfected. The frequencies from the infected groups are shown in (*J*), (*K*), and (*L*). Each data point represents one mouse. Error bars = means +/- SEM.

Supplementary Figure 15

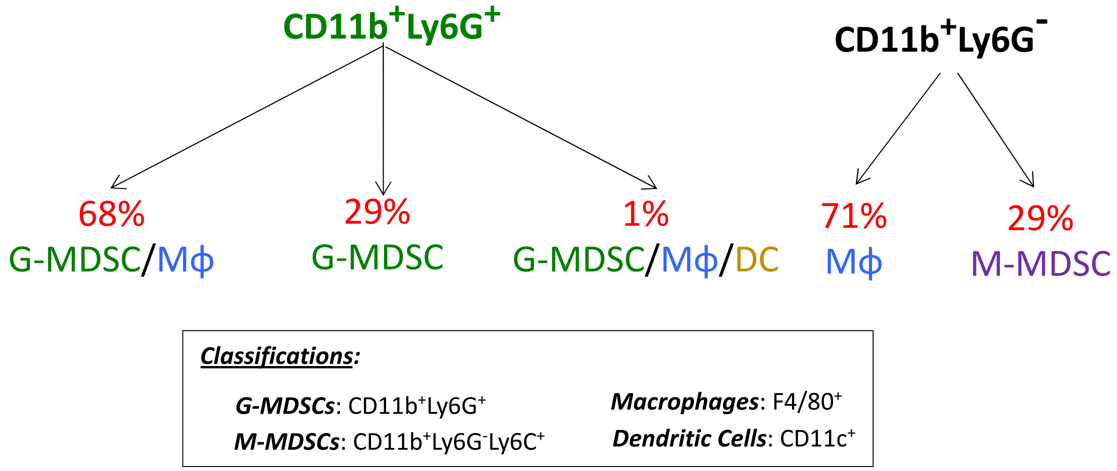

Supplementary Figure 15. Summary of the distinctness and overlap between gastric $CD11b^{+}Ly6G^{+}$ G-MDSCs, and what had been traiditonally classified as gastric $F4/80^{+}$ macrophages, and gastric $CD103^{+}CD11c^{+}$ dendritic cells based on the FACS plots and summary in Supplementary Figures 16 and 17 respectively.

Supplementary Figure 16

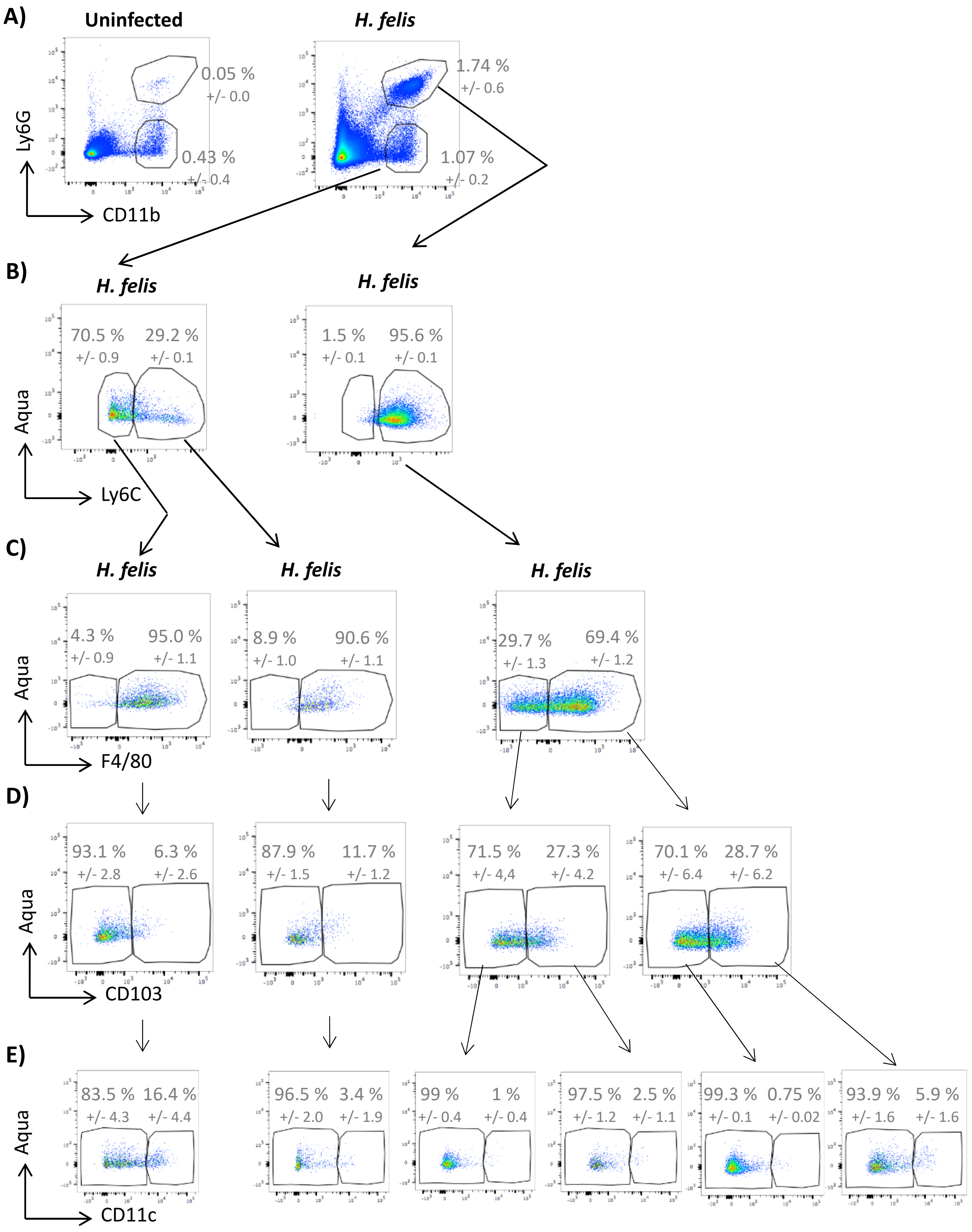

Supplementary Figure 16. Characterization of the distinctness and overlap of gastric CD11b$^+$Ly6G$^+$ G-MDSCs and gastric CD11b$^+$Ly6G$^-$ non-G-MDSC myeloid cells relative to the

expression of F4/80 (marker of macrophages), and CD103 and CD11c (markers of dendritic cells). FACS plots from *(A)* gastric $CD11b^{+}Ly6G^{+}$ G-MDSCs and gastric $CD11b^{+}Ly6G^{-}$ non-G-MDSC myeloid cells following 6mo *H. felis*-infection were gated according to *(B)* Ly6C expression, followed by *(C)* F4/80 expression, *(D)* CD103 expression, and *(E)* CD11c expression. The percentage values are indicative of the mean percentages from three 6mo *H. felis*-infected mice per group +/- SEM.

Supplementary Figure 17

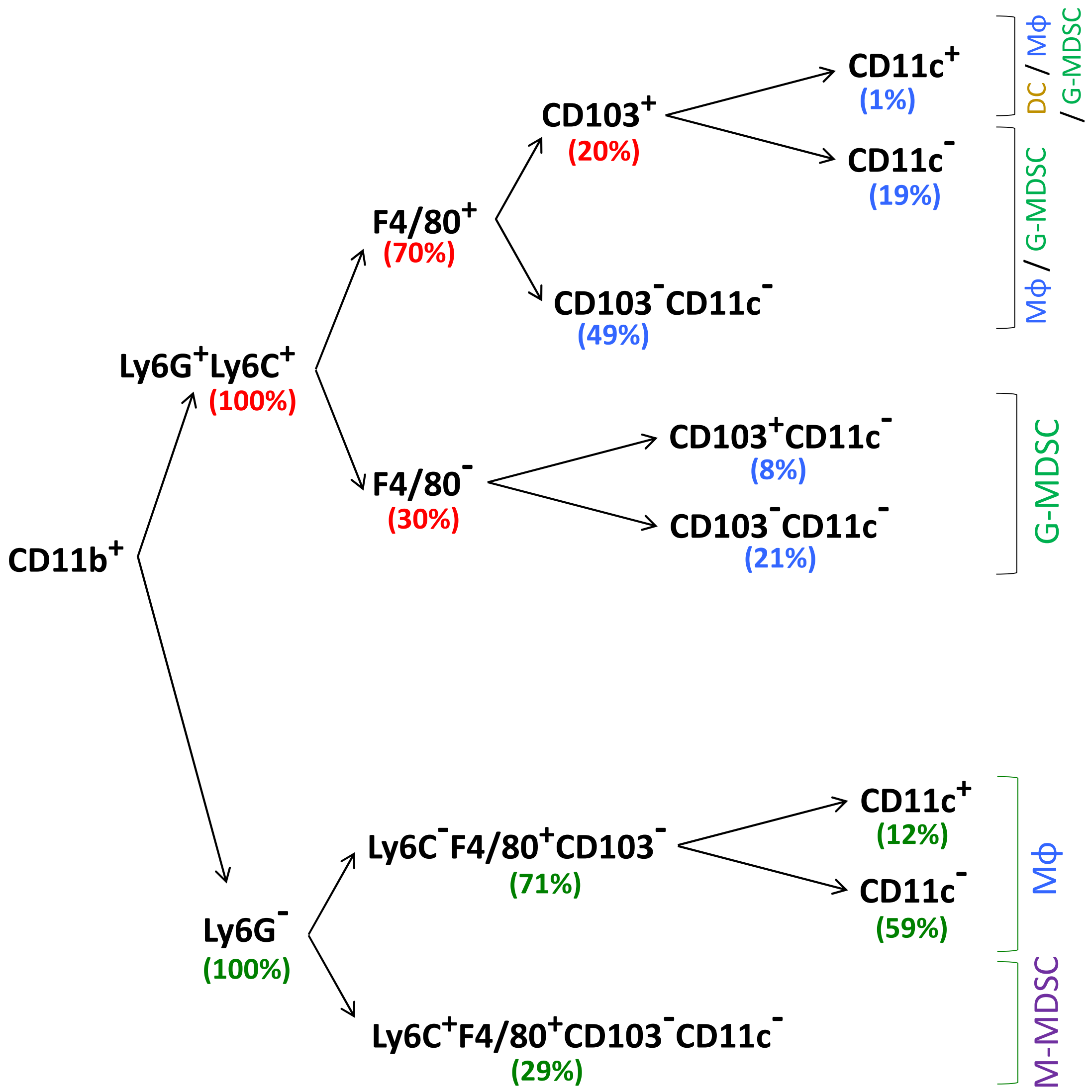

Supplementary Figure 17. Summary of the distinctness and overlap of CD11b^{+}Ly6G^{+} G-MDSCs and gastric CD11b^{+}Ly6G^{-} non-G-MDSCs relative to Ly6C, F4/80, CD103 and CD11c marker expression, based on an <u>initial</u> CD11b^{+} and Ly6G^{+} or Ly6G^{-} gating of gastric cells from <u>three</u> 6mo *H. felis*-infected mouse stomachs. The listed percentages are based on the gating in Supplementary Figure 16.
